# Temporal immunomodulation of CD4^+^ T cells by magnesium regulates osteoimmune responses in osteoporotic fracture healing

**DOI:** 10.1126/sciadv.aeb2091

**Published:** 2026-06-26

**Authors:** Jung Hun Kim, Tae Hoon Kang, SuWan Jeon, Nathaniel S. Hwang

**Affiliations:** ^1^ School of Chemical and Biological Engineering, the Institute of Chemical Processes, Seoul National University, Seoul 08826, Republic of Korea.; ^2^ Institute of Engineering Research, Seoul National University, Seoul 08826, Republic of Korea.; ^3^ Interdisciplinary Program in Bioengineering, Seoul National University, Seoul 08826, Republic of Korea.; ^4^ BioMAX/N-Bio Institute, Institute of BioEngineering, Seoul National University, Seoul 08826, Republic of Korea.

## Abstract

Osteoporotic fracture healing is impaired by dysregulated immune responses characterized by a T_H_1/M1-biased inflammatory microenvironment. In this study, we show that extracellular magnesium ions (Mg^2+^) reshape this osteo-immune niche in a dose- and time-dependent manner. Briefly, Mg^2+^ suppresses TRPM7-mediated Ca^2+^ spikes and the NFATc1-driven proinflammatory axis, thereby promoting T_H_2/M2 responses. However, sustained excess Mg^2+^ attenuates T_H_2/M2 responses by inhibiting Orai1/CaV-dependent Ca^2+^ influx and reactivating T_H_1/M1 responses through JAK-STAT1 signaling under low-calcium stimulation condition. To therapeutically use these dynamics, we engineered a bioceramic intramedullary nail (IMN) with a precisely controlled Mg^2+^ release profile, delivering Mg^2+^ in a time-phased manner. In an ovariectomized mouse fracture model, this optimized IMN reduced T_H_1/M1 of early phase proinflammatory cells, enhanced T_H_2/M2 responses during the remodeling phase, and supported coordinated immune regulation during osteoporotic fracture healing. These findings identify time-phased Mg^2+^ delivery as a strategy to mitigate excessive inflammatory responses through temporal immunomodulation of CD4^+^ T cells during osteoporotic bone healing.

## Teaser

Time-phased magnesium delivery modulates CD4^+^ T cell immunity to mitigate inflammation during osteoporotic fracture healing.

## INTRODUCTION

Osteoporotic fracture healing is often compromised not only by reduced bone mineral density (BMD) that predisposes to cortical fragility but also by a chronically T helper cell 1 (T_H_1)/T_H_17-skewed immune microenvironment and persistent M1 macrophage activity that impair coordinated bone regeneration (*1*). Postmenopausal osteoporosis of estrogen deficiency chronically activates T cells and macrophages, inducing sustained secretion of tumor necrosis factor–α (TNF-α), interleukin-1β (IL-1β), and IL-17. These proinflammatory cytokines raise receptor activator of nuclear factor kappa-Β ligand (RANKL) expression, thereby promoting osteoclastogenesis (*2*–*4*). In both ovariectomized mice and elderly patients, early-stage fracture is marked by heightened secretion of proinflammatory cytokines by activated T_H_1 cells and M1 macrophages, a key contributor to delayed union (*5*–*7*). As a result, the fracture niche evolves into an inflamed marrow cavity, where dysregulated bone remodeling characterized by an imbalanced formation-to-resorption ratio that leads to the development of mechanically fragile callus prone to secondary fracture (*8*). Therefore, material strategies that actively modulate the inflammatory microenvironment may complement structural fixation in osteoporotic fractures. This has motivated interest in intramedullary implants that can attenuate excessive early inflammation and support a transition toward a T_H_2/M2-associated reparative phase (*9*).

Magnesium ion (Mg^2+^), the fourth most abundant cation in the human body, serves as a multifunctional modulator of bone repair (*10*). Mg^2+^ promotes mesenchymal stem cell osteogenesis (*11*, *12*), up-regulates transcription factor Sp7 (SP7/Osterix) and bone matrix production (*13*), stimulates endothelial nitric oxide synthase (*14*), and activates Wnt signaling of human bone marrow stromal cells (*15*)—collectively supporting both osteogenesis and angiogenesis. Mg^2+^ also inhibits nuclear factor κB (NF-κB) (*16*), reduces TNF-α and IL-6 production (*17*), and polarizes macrophages toward the M2 phenotype (*18*), thereby suppressing osteoclast differentiation. Because of these properties, Mg-based biomaterials are increasingly studied for orthopedic application (*10*). However, limitations remain that impede clinical translation. Recent studies have reported that excess Mg^2+^ may impair late-stage bone remodeling by increasing tartrate-resistant acid phosphatase (TRAP)^+^ osteoclast numbers and hindering extracellular matrix mineralization (*19*). Furthermore, high level of Mg^2+^ can reactivate proinflammatory response (*20*). Considering the immunological environment of osteoporotic bone healing, this immune reactivation driven by Mg^2+^ could ultimately compromise optimal bone regeneration. Despite this concern, the immunological mechanisms linking Mg^2+^ exposure to bone healing outcomes remain poorly understood.

**Fig. 1.**
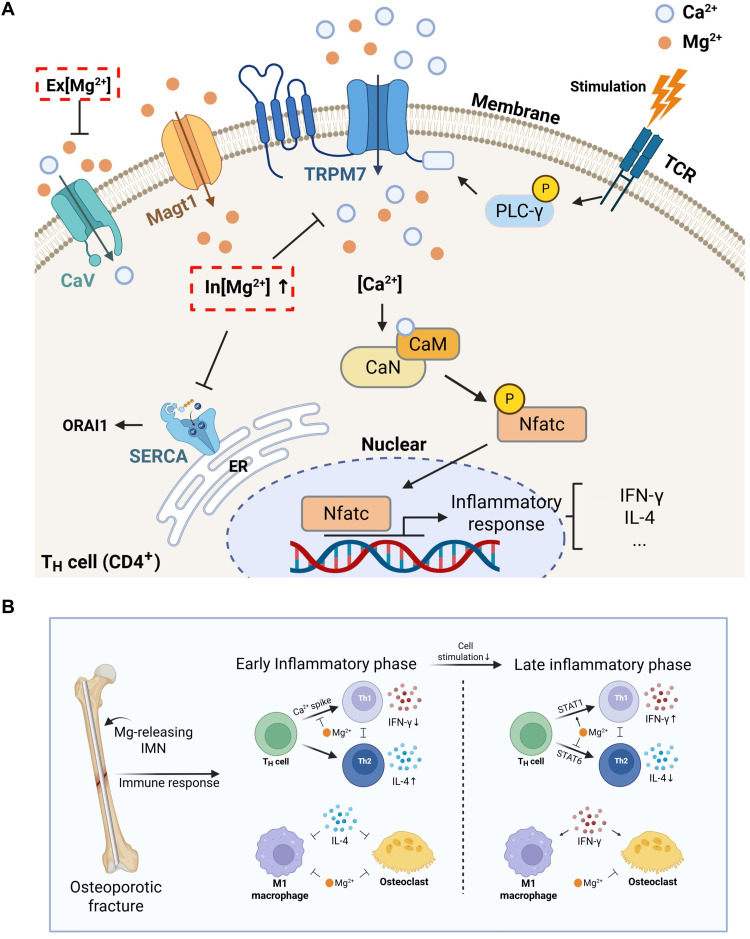
Schematic proposed mechanism of magnesium’s in vitro and in vivo actions. (**A**) Proposed cellular effects of magnesium ion [created in BioRender. Hwang, N. (2026) https://BioRender.com/6ohtoks]. (**B**) Magnesium effects to inflammatory response in osteoporotic fracture [created in BioRender. Hwang, N. (2026) https://BioRender.com/9ydtta2].

This study was designed to address this critical gap, by defining the narrow therapeutic window of Mg^2+^ and elucidate its immunological mechanism of action in osteoporotic fracture healing (Fig. 1). We show that sustained high-dose Mg^2+^ exposure reactivates the proinflammatory response, which potentially lead to delayed regeneration and increased risk of refracture. We aimed to elucidate the dose- and time-dependent effects of Mg^2+^ on CD4^+^ T cell T_H_1/T_H_2 balance, macrophage M1/M2 polarization, and osteoclast-associated inflammatory responses. Early-stage administration of Mg^2+^ (≈ 5 mM) suppresses the T_H_1/M1/osteoclast cascade by inhibiting transient receptor potential cation channel, subfamily M, member 7 (TRPM7)-mediated Ca^2+^ signaling, thereby reducing inflammatory cell infiltration. In contrast, prolonged exposure to high Mg^2+^ concentration triggers interferon-γ (IFN-γ)/signal transducer and activator of transcription 1 (STAT1)–driven T_H_1 reactivation, delays mineralization, and sustains inflammatory activity. To address this, we developed bioceramic-coated intramedullary nail (IMN) that delivers a Mg^2+^ in a precisely time-phased manner, with an early burst followed by rapid tapering. This strategy suppresses early excessive proinflammation, reduces osteoclastogenesis, and enhances bone repair quality. These findings identify finely tuned Mg^2+^ release as a key design principle for osteoimmunomodulatory implants for osteoporotic bone repair.

## RESULTS

### Excessive Mg^2+^ promotes T_H_1-associated inflammation and alters the osteoimmune environment in vivo

**Fig. 2.**
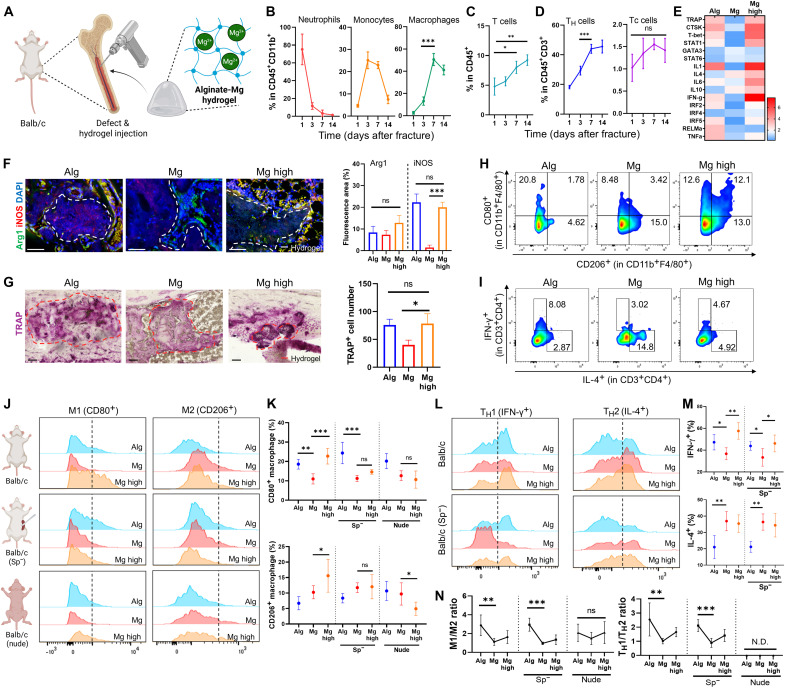
Osteo-immune responses elicited by a magnesium-releasing alginate hydrogel at the bone regeneration site. (**A**) Schematic of bone defect creation followed by Mg-alginate hydrogel injection in Balb/c [created in BioRender. Hwang, N. (2026) https://BioRender.com/td8s8zj]. (**B** to **D**) Time-dependent changes in cellular composition within the defect after hydrogel treatment (*n* = 3). (**E**) Heatmap comparing relative gene expression levels among groups (color scale represents group means, *n* = 3). (**F**) Immunofluorescence for Arg1 and iNOS around the hydrogel and quantification (scale bars, 50 μm; *n* = 3). (**G**) TRAP staining of osteoclasts surrounding the hydrogel in the femoral defect and quantification (scale bars, 100 μm; *n* = 3). (**H**) Representative FACS analysis of CD80 and CD206 (in CD11b^+^F4/80^+^) macrophages in the defect. (**I**) Representative images showing CD3^+^CD4^+^ T cells producing IL-4 (T_H_2) or IFN-γ (T_H_1) in the defect. (**J**) Representative flow cytometry histograms of CD80 (M1) and CD206 (M2) expression in Balb/c, splenectomized (Sp^−^), and nude mice. (**K**) Quantification of CD80^+^ or CD206^+^ cells as a percentage of CD11b^+^F4/80^+^ macrophages (*n* = 6 to 7). (**L**) Representative histograms of IFN-γ^+^ and IL-4^+^ T cell subsets; nude mice (T cell deficient) are omitted. (**M**) IFN-γ^+^ and IL-4^+^ cells expressed as a percentage of CD3^+^CD4^+^ T cells (*n* = 6). (**N**) M1/M2 and T_H_1/T_H_2 ratios in control, Sp^−^, and nude mice from (K) and (M) (*n* = 6 to 7; N.D., not determined). Error bars indicate mean ± SD. Statistical significance: Tukey’s multiple comparisons test; *P < 0.05, **P < 0.01, and ***P < 0.001. DAPI, 4′,6-diamidino-2-phenylindole.

To investigate the dose-dependent immunomodulatory capacity of Mg^2+^, we created femoral defect in mice and filled it with alginate hydrogels releasing ≈0 (Alg), 2.5 mM/day (Mg), or 6 mM/day (Mg high) Mg^2+^ over the first postoperative week (Fig. 2A and fig. S1A) (*19*). Flow cytometric profiling of cells (fig. S1, B and C) harvested from the defect showed that the acute inflammatory phase (day 1) of the defect was dominated by neutrophils (CD11b^+^Ly6C^+^Ly6G^−^), whereas at days 3 and 7, macrophages (CD11b^+^F4/80^+^), T cells (CD45^+^CD3^+^), and helper T cells (CD45^+^CD3^+^CD4^+^, T_H_ cells) were the main recruited populations, and natural killer cells (CD45^+^CD3^−^NK1.1^+^), B cells (CD45^+^CD3^−^B220^+^), and cytotoxic T cells (CD45^+^CD3^+^CD8^+^, T_c_ cells) showed little change (Fig. 2, B to D, and fig. S1D). These data indicate that local biomaterial-induced immune responses in bone defects are primarily orchestrated by macrophages and T_H_ cells.

On postoperative day 7, the Mg high group exhibited up-regulated proinflammatory gene (*IL-1, IFN-*γ*, TNF-*α*, etc.*) expression than the Mg group (Fig. 2E). Also, elevated numbers of inducible nitric oxide synthase (iNOS)–positive cells in immunostaining and a greater number of TRAP-positive osteoclasts were observed, indicating enhanced proinflammatory response and osteoclastic activation under high Mg^2+^ exposure (Fig. 2, F and G). These observations were further investigated by flow cytometry. On day 3, M1 (CD11b^+^F4/80^+^CD80^+^) and M2 (CD11b^+^F4/80^+^CD206^+^) macrophages showed that higher magnesium concentrations were associated with a decrease in M1 cells and an increase in M2 cells (fig. S2A). By day 7, however, the proportion of M1 macrophages in the Mg high group increased substantially compared to the Mg group (Fig. 2H). Furthermore, examination of T_H_1 (CD3^+^CD4^+^IFN-γ^+^) and T_H_2 (CD3^+^CD4^+^IL-4^+^) cells revealed that the Mg high group exhibited a higher proportion of T_H_1 cells and lowered T_H_2 (Fig. 2I).

To dissect the role of T cells in this immune skewing, we applied the same hydrogel model using splenectomized (Sp^−^) and T cell–deficient (nude) mice that exhibit reduced or absent of blood circulating T cells (fig. S2B). On day 7, the M1 and M2 polarization induced by Mg high was attenuated in both Sp^−^ and nude mice than Balb/c control (Fig. 2, J and K). Notably, the frequency of IFN-γ^+^ T_H_1 cells remained elevated in the Mg high–treated Sp^−^ mice, same as the Balb/c controls (Fig. 2, L and M). M1/M2 and T_H_1/T_H_2 ratios in Sp^−^ mice resembled Balb/c controls. Although both M1 and M2 macrophage populations were reduced in nude mice, the M1/M2 ratio did not exhibit Mg^2+^-dependent changes (Fig. 2N). These findings suggest that Mg^2+^-dependent modulation of macrophage polarization requires the presence of T cells, highlighting the importance of T cells in mediating Mg^2+^-driven immune regulation within the osteoimmune environment.

Together, these results suggest that Mg^2+^ exerts anti-inflammatory effects during the early inflammatory phase but promotes a T_H_1-dominant immune response when present at excessive levels. The absence of Mg^2+^-dependent changes in macrophage polarization in nude mice further indicates that T cells are required for this immunomodulatory effect. On the basis of these observations, we next investigated the molecular mechanisms by which excess Mg^2+^ promotes T_H_1-associated immune responses.

### Under strong Ca^2+^ stimulation, extracellular Mg^2+^ suppresses the T_H_1 differentiation

**Fig. 3.**
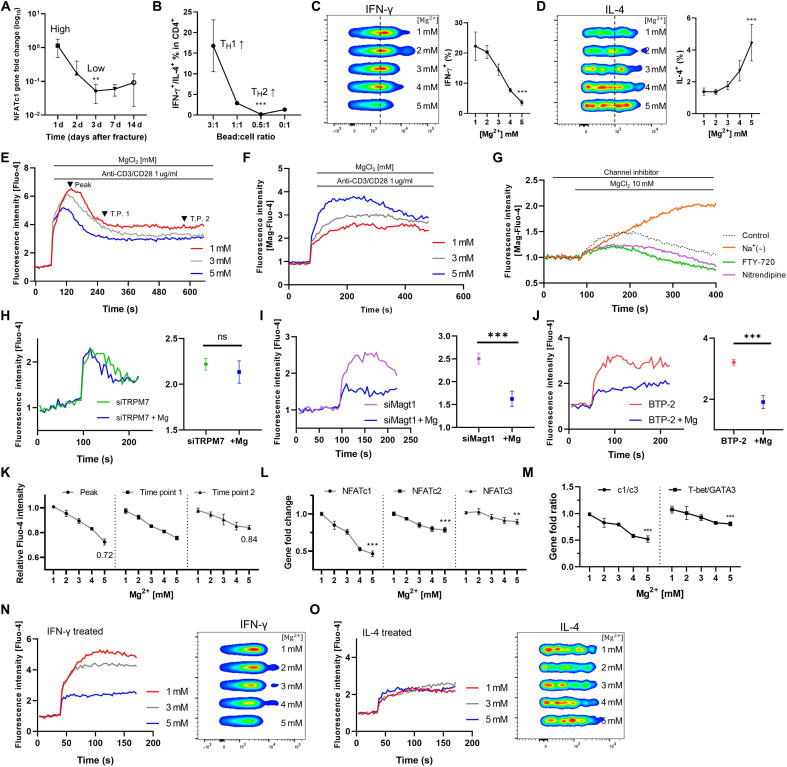
Strong Ca^2+^ stimulation skews CD4^+^ T cells toward T_H_1 phenotype, and extracellular Mg^2+^ reduces this bias. (**A**) Fold change of Nfatc1 transcripts in a bone defect site without any implanted biomaterials (*n* = 3). d, days. (**B**) Ratio of IFN-γ to IL-4 production in splenic CD4^+^ T cells cultured for 3 days with anti-CD3/CD28 beads at the indicated bead-to-cell (B:C) ratios (*n* = 3). (**C** and **D**) With B:C = 3:1, representative concatenated flow cytometry plots and quantification of IFN-γ^+^ and IL-4^+^ cells at B:C = 3:1 under increasing Mg^**2**+^ concentrations (*n* = 3). (**E**) Time course of Fluo-4 Ca^**2**+^ fluorescence in CD4^+^ T cells stimulated at B:C = 3:1 while stepwise raising extracellular Mg^**2**+^. (**F**) Mag-Fluo-4 imaging of intracellular Mg^**2**+^ at B:C = 3:1. (**G**) Mag-Fluo-4 in cells treated with 10 mM Mg^**2**+^ with Mg^**2**+^ transporter inhibitor or natrium ion depleted. (**H** and **I**) Fluo-4 Ca^**2**+^ imaging and mean peak values after Mg^**2**+^ addition in cells transfected with siTRPM7 or siMagt1 (*n* = 3). (**J**) Fluo-4 Ca^**2**+^ traces and mean peak values with or without the SOCE inhibitor BTP-2 during Mg^**2**+^ treatment (*n* = 3). (**K**) Quantitative analysis of the Ca^**2**+^ peak height and two subsequent time points from (E) (*n* = 3). (**L** and **M**) Expression of *NFATc1*, *NFATc2*, and *NFATc3*, and *NFATc1/NFATc3* and *T-bet/Gata3* ratios after 3 days in graded Mg^**2**+^ (*n* = 3). (**N** and **O**) Representative Fluo-4 imaging and concatenated FACS plots of CD4^+^ cells pretreated for 3 days with IFN-γ or IL-4 and restimulated at B:C = 3:1. Error bars represent mean ± SD. Significance was determined by Tukey’s multiple comparisons test (*P < 0.05, **P < 0.01, and ***P < 0.001).

Inflammatory signaling in bone defects from the foreign body response to biomaterial implants begins with a burst of intense cellular activation that wanes over time (*21*, *22*). Because the expression of *nuclear factor of activated T cell* (*NFAT*) genes is Ca^2+^ dependent, their progressive down-regulation (*NFATc1, NFATc2, and NFATc3*) in the defect indicates that cumulative Ca^2+^ signaling falls as healing proceeds (Fig. 3A and fig. S3A). To mimic this osteo-immune environment ex vivo, isolated splenic CD4^+^ T cells (CD4^+^CD8^−^NK1.1^−^B220^−^) were stimulated with anti-CD3/CD28–coated beads at graded bead-to-cell ratios—a well-established proxy for tuning T cell receptor (TCR)/CD28–driven Ca^2+^ oscillations. MgCl_2_ was added at 1 to 5 mM, a range selected on the basis of Mg^**2+**^ concentrations observed in vivo, and higher doses (>5 mM) reduced viable cell numbers (fig. S3B). Chloride control added as N-methyl-D-glucammonium chloride (NMDG-Cl) had no effect on the T_H_1/T_H_2 transcription factor ratio (*T-box expressed in T cells, T-bet/GATA-binding protein 3, GATA3*), confirming that MgCl_2_ acts via extracellular Mg^2+^ rather than Cl^−^ (fig. S3C).

High bead density (bead to cell, B:C = 3:1) produced a strong intracellular Ca^2+^ drive that favored T_H_1 differentiation in line with previous reports (Fig. 3B) (*23*, *24*). Under strong stimulation, Mg^2+^ suppressed T_H_1 cells (CD4^+^IFN-γ^+^) and dose-dependently enhanced T_H_2 cells (CD4^+^IL-4^+^) (Fig. 3, C and D). Fluo-4 imaging showed that intracellular Ca^2+^ fell as extracellular Mg^2+^ rose (Fig. 3E). Mag-Fluo-4 demonstrated the converse—higher Mg^2+^ influx with rising extracellular Mg^2+^ (Fig. 3F). Mg^2+^ transporters TRPM7 and Magt1 were identified as the principal Mg^2+^ entry routes (*25*, *26*). Blocking Mg^2+^ transport with the TRPM7 inhibitor FTY-720 or the Magt1 inhibitor nitrendipine reduced Mg^2+^ influx, highlighting both channels as major entry routes, whereas Na^+^ removal increased Mg^2+^ accumulation (Fig. 3G). Knockdown of TRPM7 abolished Mg-induced Ca^2+^ suppression (Fig. 3H), whereas Magt1knockdown or Orai1 blockade (BTP-2) did not (Fig. 3, I and J). Thus, elevated extracellular Mg^2+^ enters via TRPM7/Magt1; the resulting rise in intracellular [Mg^2+^]_i_ feeds back to inhibit TRPM7-mediated Ca^2+^ entry under strong stimulatory condition. The Ca^2+^-calcineurin-NFAT axis mirrored these fluxes: The initial Ca^2+^ peak correlated with *NFATc1*, whereas later Ca^2+^ levels tracked with *NFATc2* and *NFATc3* (Fig. 3, K and L). Immunocytochemistry confirmed differential nuclear translocation at 5 mM Mg^2+^ (NFATc3 > NFATc1; fig. S4A). *T-bet* and *GATA3* expression fell with rising Mg^2+^ and paralleled the *NFATc1*/*NFATc3* ratio (Fig. 3M and fig. S4B). To probe functional relevance, cells differentiated for 3 days with IFN-γ or IL-4 were restimulated by anti-CD3/CD28 bead for Ca^2+^ imaging. Mg^2+^ dampened the Ca^2+^ surge only in IFN-γ–conditioned cells that exhibit high TRPM7 expression; IL-4–conditioned cells were unaffected (Fig. 3, N and O, and fig. S4C). In sum, a strong Ca^2+^ drive steers T_H_0 cells toward the proinflammatory T_H_1 lineage. Extracellular Mg^2+^, by entering through TRPM7 and Magt1, inhibits Ca^2+^ entry and blocks T_H_1 commitment. This restriction simultaneously enlarges the T_H_2 compartment, thereby tipping the balance toward anti-inflammation within the therapeutic Mg^2+^ window.

### Changing the intensity of Ca^2+^ stimulation alters the immunological effect of Mg^2+^ in T_H_ differentiation

**Fig. 4.**
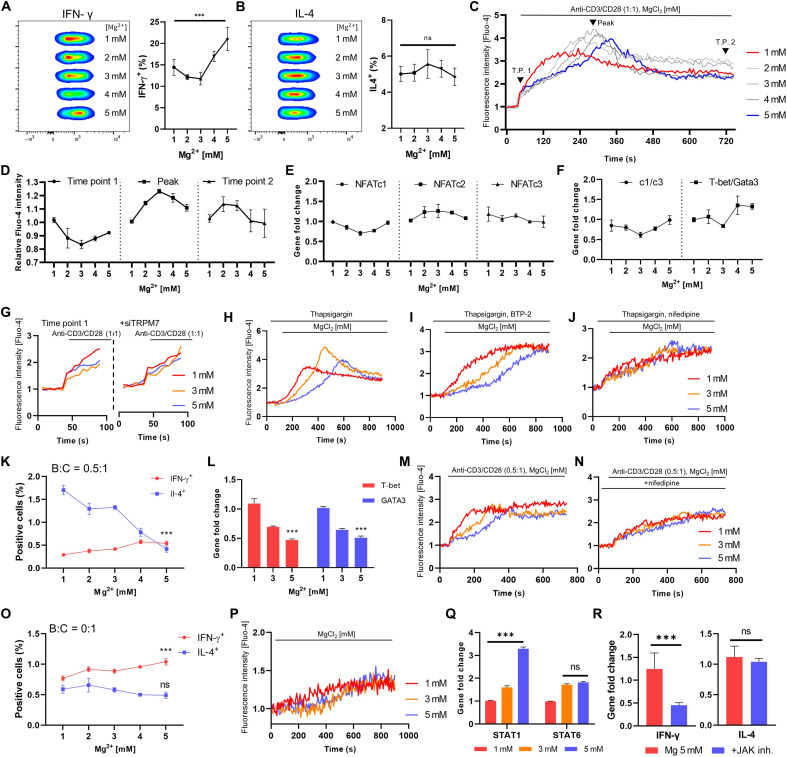
Extracellular Mg^2+^ reshapes Ca^2+^ flux signatures and effector bias of CD4^+^ T cells under moderate or low TCR stimulation. (**A** and **B**) Representative concatenated FACS plots and quantification (A) of IFN-γ^+^ (T_H_1) and (B) IL-4^+^ (T_H_2) cells after 3-day culture in graded Mg^**2**+^ with moderate stimulation (B:C = 1:1) (*n* = 3). (**C**) Fluo-4 Ca^**2**+^ imaging of CD4^+^ T cells cultured in B:C = 1:1; T.P. 1 is early peak; T.P. 2 denotes postpeak plateaus. (**D**) T.P. 1, mean peak, and T.P. 2 quantification from Fluo-4 Ca^**2**+^ imaging (C) (*n* = 3). (**E** and **F**) Relative expression of *NFATc1*, *NFATc2*, and *NFATc3*, and *NFATc1*/*NFATc3* and *T-bet*/*Gata3* ratios under B:C = 1:1 conditions with Mg^**2**+^ treatment (*n* = 3). (**G**) Early Ca^**2**+^ spike triggered by anti-CD3/CD28 beads: comparison of siTRPM7-transfected cells versus Mg^**2**+^ supplementation. (**H** to **J**) SOCE-driven Ca^**2**+^ imaging of CD4^+^ T cells upon (H) Tg (25 nM), (I) BTP-2 (SOCE blocker), (J) nifedipine (L-type Ca^**2**+^ channel blocker), and MgCl_2_. (**K**) Frequencies of IFN-γ^+^ and IL-4^+^ cells after 3 days in low stimulation (B:C = 0.5:1) (*n* = 3). (**L**) T-bet and Gata3 transcript levels under the B:C = 0.5:1 condition after 3 days (*n* = 3). (**M** and **N**) Ca^**2**+^ flux profiles across Mg^**2**+^ doses in B:C = 0.5:1 condition and the effect of nifedipine (N) with Mg^**2**+^. (**O**) Percentages of IFN-γ^+^ and IL-4^+^ cells in no TCR stimulation cultures (*n* = 3). (**P**) Representative Ca^**2**+^ imaging of unstimulated cells. (**Q**) Mg^**2**+^ dose–dependent modulation of *STAT1* and *STAT6* mRNA (*n* = 3). (**R**) Effect of a JAK inhibitor on Mg^**2**+^-induced *IFN-*γ and *IL-4* transcripts (*n* = 3). Error bars denote mean ± SD. Statistical significance: Tukey’s multiple comparisons test (***P < 0.001).

When CD4^+^ T cells were activated with a B:C = 1:1 of moderate intensity, raising extracellular Mg^2+^ above than 3 mM inverted the differentiation pattern, the T_H_1 fraction rose while T_H_2 fell (Fig. 4, A and B). Ca^2+^ imaging showed a small early rise [time point 1 (T.P.1)] followed by a slow ramp to a single peak and a late plateau (T.P.2) (Fig. 4C). Fluorescence at each phase had a correlation with NFAT isoforms—T.P.1 with *NFATc1*, the peak with *NFATc2*, and T.P.2 with *NFATc3* (Fig. 4, D and E). Consequently, the *NFATc1*/*NFATc3* ratio paralleled the T-bet/GATA3 ratio (Fig. 4F and fig. S5A). Unlike the strong-stimulus (3:1) setting, extracellular Mg^2+^ had little effect on the initial Ca^2+^ influx, TRPM7 knockdown barely changed the Ca^2+^ trace, and Mg^2+^ influx did not correlate with the MgCl_2_ dose (Fig. 4G and fig. S5B). Thus, the early Ca^2+^ entry at this stimulus level is independent of TRPM7, and it could be another channel or endoplasmic reticulum (ER) calcium release. Because a subsequent phase of sustained Ca^2+^ entry is attributed to store-operated Ca^2+^ entry (SOCE) (*27*), we studied T cells with thapsigargin (Tg). The Ca^2+^ trace had similar tendency with the bead-induced curve and varied with Mg^2+^ in the same way (Fig. 4H). Intracellular Mg^2+^ fluorescence coincided with the Ca^2+^ Fluo-4 peak (fig. S5C), suggesting that intracellular Mg^2+^ alleviates SOCE stress. The SOCE blocker BTP-2 abolished the Ca^2+^ peak, yet Mg^2+^ still modulated the Ca^2+^ entry (Fig. 4I), whereas the voltage-gated calcium channel (CaV) inhibitor nifedipine abolished this Mg-dependent rate modulation (Fig. 4J). Together, these data indicate that, at moderate stimulation, the dominant pathway is SOCE through Orai1 and voltage-gated CaV channels, not TRPM7. In addition, Mg^2+^ likely reduces SOCE stress and decreases membrane depolarization.

Under B:C = 0.5:1 ratio of weak activation, higher Mg^2+^ produced a modest increase in T_H_1 cells and a decrease in T_H_2 cells (Fig. 4K). T-bet and GATA3 transcripts declined dose-dependently (Fig. 4L). Ca^2+^ influx again decreased with rising Mg^2+^, but this effect vanished with nifedipine, to a CaV channel inhibition (Fig. 4, M and N). In the absence of anti-CD3/CD28 bead engagement, T_H_1 frequency rose slightly, and T_H_2 remained unchanged (Fig. 4O). Ca^2+^ signals were negligible, and Mg^2+^ had virtually no effect on Ca^2+^ fluxes (Fig. 4P). Contrary to our initial expectation, adenosine triphosphate (ATP)/adenosine diphosphate ratios and *mechanistic target of rapamycin* (*mTOR*) transcripts decreased, whereas STAT genes—especially *STAT1* relative to *STAT6*—increased with Mg^2+^ (Fig. 4Q and fig. S5D). Cotreatment with a Janus kinase (JAK) inhibitor and 5 mM Mg^2+^ reduced *IFN-*γ expression (Fig. 4R), indicating that Mg^2+^ activates JAK-STAT signaling with a bias toward IFN-γ/STAT1, and it also explains the T_H_1 elevation under low stimulus condition. However, the cell number fell by ~50% at 5 mM Mg^2+^ in Ca^2+^ stimulus-free culture, so lineage data in this setting reflect the expression profile of the surviving cell population (fig. S5E).

### Magnesium inhibits macrophage polarization and osteoclast differentiation

**Fig. 5.**
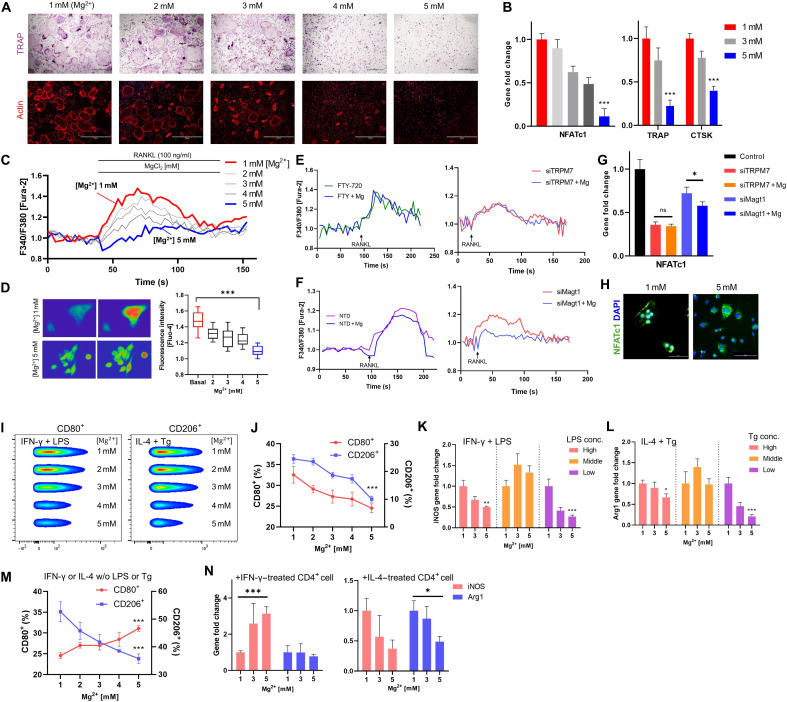
Magnesium attenuates osteoclast differentiation and tunes macrophage M1/M2 polarization. (**A**) Representative TRAP/phalloidin-actin images of osteoclasts cultured 5 days with RANKL (100 ng ml^−**1**^) plus the indicated Mg^**2**+^ concentrations. Scale bars, 1000 μm. (**B**) Mg^**2**+^ dose–dependent expression of osteoclastic genes (*n* = 3). (**C**) Fura-2 Ca^**2**+^ imaging of preosteoclasts during RANKL stimulation with Mg^**2**+^, representative traces (*n* = 3). (**D**) Fluo-4 Ca^**2**+^ imaging of preosteoclasts with RANKL stimulation at Mg^**2**+^ 1 or 5 mM condition, representative images and quantification (*n* = 3). (**E**) Effect of FTY-720 or siTRPM7 on RANKL-induced Ca^**2**+^ flux in preosteoclasts. (**F**) Ca^**2**+^ flux in preosteoclasts treated with nitrendipine or siMAGT1. (**G**) Day 5 mRNA levels of osteoclast markers after siTRPM7 or siMAGT1 transfection (*n* = 3). (**H**) Immunofluorescence showing that Mg^**2**+^ reduces nuclear translocation of NFATc1. Scale bars, 50 μm. (**I**) Concatenated FACS plots of M1 (CD11b^+^F4/80^+^CD80^+^) and M2 (CD11b^+^F4/80^+^CD206^+^) phenotypes in macrophages treated 1 day with LPS/IFN-γ or Tg/IL-4. (**J**) Quantification of M1 and M2 cells as a percentage of F4/80^+^ macrophages with varying Mg^**2**+^ (*n* = 3). (**K**) iNOS transcript levels after LPS at 100, 25, or 10 ng ml^−**1**^ with varying Mg^**2**+^ (*n* = 3). (**L**) Arg1 transcripts after Tg at 100, 50, or 10 nM with varying Mg^**2**+^ (*n* = 3). (**M**) Mg^**2**+^ dose effects on M1/M2 marker expression in macrophages polarized with IFN-γ or IL-4 (*n* = 3). (**N**) *iNOS* and *Arg1* mRNA in M0 macrophages 3 days after reexposure to conditioned CD4^+^ T cells previously cultured with IFN-γ + Mg^**2**+^ or IL-4 + Mg^**2**+^. Error bars represent mean ± SD. Significance: Tukey’s multiple comparisons test (*P < 0.05 and ***P < 0.001).

Numerous studies report that Mg^2+^ attenuates M1 macrophage activation and inhibits osteoclastogenesis, yet the underlying mechanism remains unclear. We therefore tested whether the Ca^2+^-modulating logic established for T_H_ cells applies equally to macrophages and osteoclasts differentiated from bone marrow monocytes (BMMs) in the Mg^2+^-conditioned environment. BMMs were isolated, cultured with macrophage colony-stimulating factor (M-CSF) ± RANKL, and exposed to graded MgCl_2_ (1 to 5 mM). Cell viability was unaffected (fig. S6A). Mg^2+^ suppressed osteoclastogenesis in a dose-dependent manner; TRAP-positive multinuclear cells and actin ring-forming cells declined; and *NFATc1*, *TRAP*, and *CTSK* mRNAs fell accordingly (Fig. 5, A and B). In addition, NMDG-Cl had no effect on osteoclast differentiation (fig. S6B).

RANKL-induced Ca^2+^ signaling—essential for osteoclast formation—was weakened by Mg^2+^ regardless of RANKL dose (Fig. 5, C and D, and fig. S7A). As in T cells, Mg^2+^ influx occurred via TRPM7 and Magt1 (fig. S7B). Blocking TRPM7 (FTY-720 or siTRPM7) abolished Mg-dependent Ca^2+^ suppression (Fig. 5E), while Magt1 knockdown (nitrendipine or siMagt1) only partially attenuated Ca^2+^ flux (Fig. 5F). NFATc1 mRNA showed similar tendencies to these Ca^2+^ traces (Fig. 5G). Reduced Ca^2+^ entry lowered calcineurin activity, limited NFATc1 nuclear translocation, and down-regulated fusion genes (*Dcstamp*, *Ocstamp*, and *Atp6v0d2*), directly blocking osteoclast differentiation (Fig. 5H and fig. S7, C to E). Supplementing IFN-γ or IL-4 shifted the absolute level of osteoclastogenesis but did not alter the Mg-inhibiting of tendency (fig. S7F).

M0 macrophages were polarized with IFN-γ or IL-4 and assessed by CD80 (M1) or CD206 (M2). With strong Ca^2+^ stimulation—lipopolysaccharide (LPS) or Tg—Mg^2+^ reduced both M1 and M2 polarization (Fig. 5, I and J) and lowered *iNOS* and *Arg1* transcripts (Fig. 5, K and L). Without exogenous supplement, Mg^2+^ also reduced polarization (fig. S7G). Ca^2+^ imaging confirmed robust fluxes in the LPS/Tg groups that were suppressed by Mg^2+^ (fig. S7H). FTY-720 diminished this suppression in LPS-treated cells, whereas nitrendipine only partly suppressed. Neither drug altered the Tg response notably (fig. S7I). In contrast, in the absence of Ca^2+^ stimulation agents, IFN-γ or IL-4 alone did not elevate Ca^2+^ influx. In addition, increasing extracellular Mg^2+^ raised the M1 fraction while lowering M2 (Fig. 5M). Coculturing IFN-γ– or IL-4–conditioned CD4^+^ T cells with M0 macrophages revealed that strong *IFN-*γ expression heightened M1 bias, indicating that T_H_1-derived cytokines override Mg-mediated suppression of polarization (Fig. 5N). In conjunction with our previous demonstration that under low Ca^2+^ drive, Mg^2+^ pushes T_H_0 toward T_H_1 and increases *STAT1* expression, and Mg^2+^ shifts the balance toward M1 in a cytokine-rich environment. These observations highlight the need to modulate the time-phased Mg^2+^ release profile throughout the inflammatory phase.

### Time-phased Mg^2+^-releasing IMN harmonizes early anti-inflammatory and late proregenerative immunity in the OVX model

We aimed to create an optimized form of Mg^2+^-controlled release implant for osteoporotic fracture model and verify the effect of Mg^2+^ on immune cells as described above. To create an osteoporotic model, ovariectomy (OVX) was performed, and the bone condition and the inflammatory state of femur bone were evaluated after 8 weeks. The bones of OVX mice showed lower bone volume and bone density than the non-ovary removal mice group (sham) on micro–computed tomography (micro-CT), indicating a typical osteoporotic state (fig. S8, A and B). In addition, the inflammatory factors, M1/M2, and T_H_1/T_H_2 balance were investigated in bone marrow isolated from osteoporotic femur bones. Compared with the sham group, bone marrow cells showed higher gene levels related to proinflammatory cytokines, and the M1/M2 and T_H_1/T_H_2 ratios in the OVX group were higher than those in the sham group (fig. S8, C to E). In the OVX model, the inflammatory phase of osteoporosis and the risk of refracture were investigated after fracture. All fractured mice were stabilized with an IMN, and healing was monitored. OVX fractures showed markedly higher proinflammatory cytokine transcripts than age-matched BALB/c fractures in the inflammatory stage (day 3), and the level of IL-4, which can accelerate bone healing in the late inflammatory phase (day 7), remained low, and the T_H_1/T_H_2 ratio stayed skewed toward T_H_1 (fig. S8, F to H). Persistently elevated inflammatory cells are known to slow osteogenesis and compromise callus quality. In the regenerated femur, the force required for refracture was inversely correlated with the early inflammatory load (fig. S8I).

**Fig. 6.**
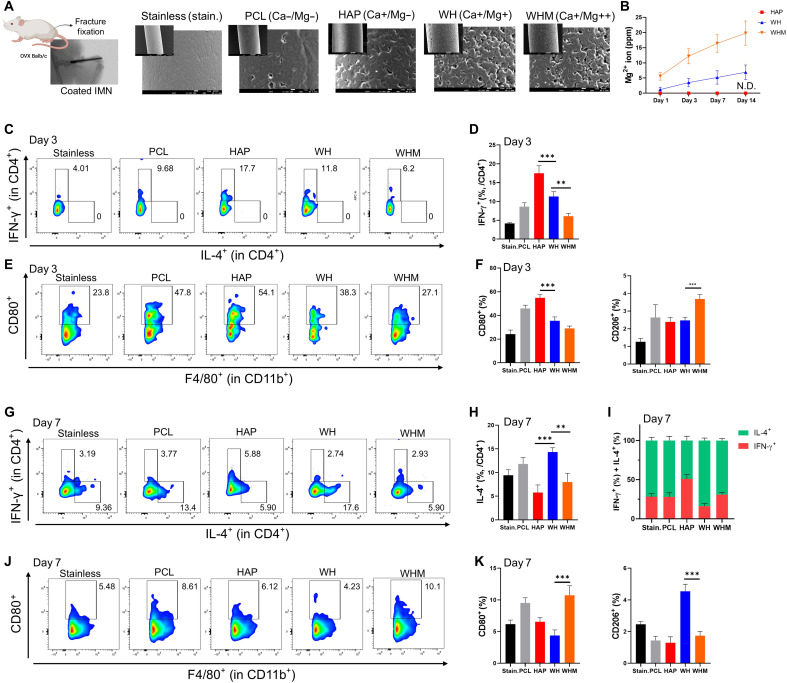
Immunomodulatory capacity of magnesium-incorporated bioceramic IMNs at a fracture site. (**A**) X-ray images of osteoporotic (OVX) Balb/c femoral fractures fixed with IMNs; HAP, WH, or magnesium-doped whitlockite (WH-Mg; WHM) IMNs, alongside SEM micrographs of each IMN surface; symbols “+” and “−” denote relative ion release characteristic. (**B**) Cumulative Mg^**2**+^ released from each IMN type into 1 ml of deionized (DI) water expressed in parts per million (ppm; *n* = 3). (**C** and **E**) Representative FACS plots of T_H_1, T_H_2, and M1 populations in bone marrow cells 3 days postfracture. (**D**) Frequency of IFN-γ^+^CD4^+^ T cells at day 3 (*n* = 5). (**F**) M1 and M2 macrophage proportions at day 3 (*n* = 5). (**G** and **J**) Day 7 FACS plots for T_H_1/T_H_2 and M1 subsets. (**H**) Proportion of IL-4^+^ T cells at day 7 (*n* = 5). (**I**) Day 7 fraction of IFN-γ^+^ and IL-4^+^ T cells (*n* = 5). (**K**) Day 7 M1 and M2 fractions expressed as percentage of F4/80^+^ cells (*n* = 5). Error bars indicate mean ± SD; Tukey’s multiple comparisons test (**P < 0.01 and ***P < 0.001).

Where high-Mg^2+^ release, under strong Ca^2+^ stimulation, suppressed T_H_1/M1, the continued excess Mg^2+^ impeded T_H_2/M2-driven repair once Ca^2+^ signaling waned while activating T_H_1 and M1, ultimately hindering regeneration. We engineered an IMN that delivers Mg^2+^ rapidly at early days but tapers thereafter. To test these dynamics, five IMN groups were fabricated composed of stainless steel, poly-caprolactone (PCL), hydroxyapatite [HAP; Ca_10_(PO_4_)_6_(OH)_2_], whitlockite [WH; Ca_18_Mg_2_(HPO_4_)_2_(PO_4_)_12_], and magnesium-doped whitlockite (WHM) (Fig. 6A). Stainless and PCL released no ions, served as a control. The Ca phosphate materials (HAP, WH, and WHM) were engineered for controlled Ca^2+^ and Mg^2+^ release. No mechanical or topographic differences were observed between the ion-releasing groups (fig. S9). Assuming a 10 μl of medullary volume, calculated local Mg^2+^ concentrations fell from day 1 to day 7 as follows: HAP, no Mg^2+^; WH, 4.9 → 1.7 mM; WHM, 23 → 4.3 mM (Fig. 6B and fig. S10A).

IMNs produced distinct immune profiles when implanted into osteoporotic fractures. The proportion of recruited CD11b^+^F4/80^+^ macrophages and CD3^+^CD4^+^ at this early stage did not show major differences among the groups (fig. S10B). At early phase (day 3), groups with greater Mg^2+^ release showed fewer T_H_1 (HAP > WH > WHM) and M1 (HAP > WH > WHM) cells, whereas M2 cells increased (WHM > WH > HAP), and T_H_2 cells were scarcely detectable (Fig. 6, C to F, and fig. S10C). At late inflammatory phase (day 7), the WHM group exhibited a higher proportion of T_H_1 cells and a lower of T_H_2 cells than the WH group (Fig. 6, G and H). Also, the calculated T_H_1 percentage appeared higher in WHM compared to WH when expressed as a fraction of total CD4^+^ T cells (Fig. 6I). Consistent with this heightened T_H_1-skewed environment, CD80^+^ M1 macrophages were also more abundant in the WHM group than in either the HAP or WH group and showed less M2 than WH (Fig. 6, J and K, and fig. S10D). By week 2, near-complete callus bridging of marrow cavity was observed, leaving insufficient cells for further fluorescence-activated cell sorting (FACS). Gene profiling of fracture tissue revealed broadly elevated immune-gene expression in WHM, notably TNF-α, IL-1, and IL-6 (fig. S10E). These data show that early high-Mg^2+^/late low-Mg^2+^ release (WH) best aligns with the therapeutic orthopedical window to achieve the most favorable immuno-regenerative balance, whereas continuous high Mg^2+^ (WHM) reignites inflammation once Ca^2+^ signaling subsides.

### WH IMN supports coordinated structural reconstruction and immune regulation during osteoporotic bone healing

**Fig. 7.**
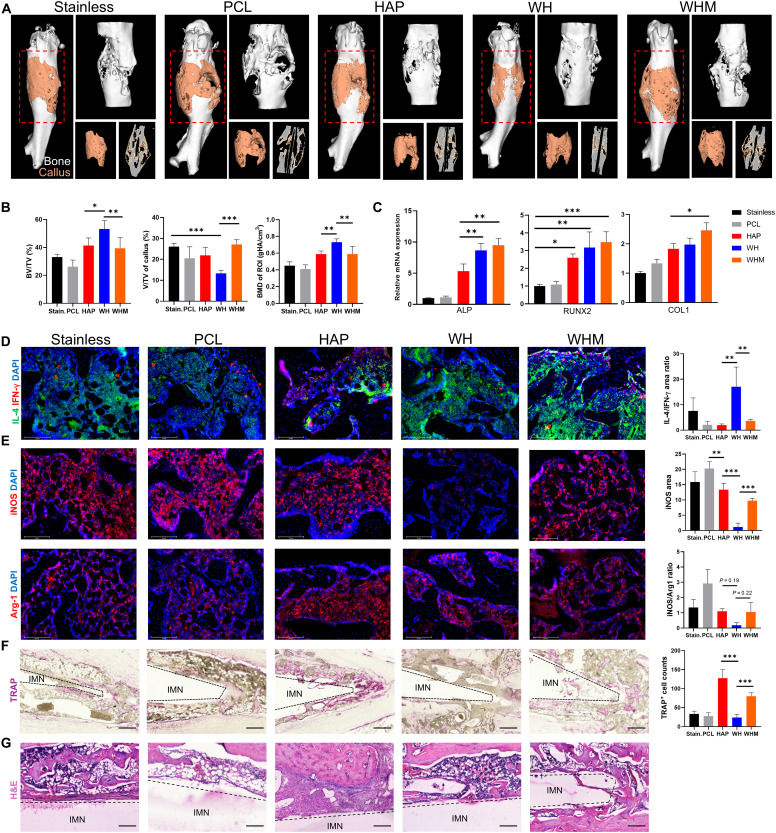
WH IMN enhances bone volume and accelerates callus remodeling. (**A**) Four-week postfracture micro-CT 3D reconstructions: cortical bone (white) and residual callus (orange), The red line box indicates the region of interest (ROI) used for the separated views of cortical bone, residual callus, and cross-section. (**B**) Quantitative BV/TV, callus V/TV, and BMD derived from micro-CT data (*n* = 5). (**C**) Osteogenic gene expression in fracture tissue (*n* = 3). (**D**) Immunofluorescence for IL-4 and IFN-γ in callus; bar graph shows IFN-γ^+^/IL-4^+^ area ratio (*n* = 4). (**E**) iNOS and Arg1 staining in callus with quantified iNOS areas and iNOS/Arg1 ratios (*n* = 4). (**F**) TRAP staining adjacent to IMN surfaces with quantification (*n* = 4). (**G**) Representative H&E sections of marrow surrounding each IMN. Error bars indicate mean ± SD. Scale bars, 125 μm. Tukey’s multiple comparisons test (*P < 0.05, **P < 0.01, and ***P < 0.001).

We evaluated the immuno-regenerative capacity of each IMN, 4 weeks after implantation. Three-dimensional (3D) reconstructed micro-CT datasets showed visual separation of newly formed bone (white) from the maturing callus (bright orange). Among all groups, the WH nail showed the thinnest callus volume and rapid reestablishment of the native near-cylindrical diaphysis (Fig. 7A). Quantitative parameters—trabecular bone volume fraction (BV/TV), callus volume fraction (callus V/TV), and BMD—together with osteogenic gene expression indicated that WHM nail showed higher osteogenic gene expression than the WH group, but the increase was nonsignificant (ALP, RUNX2, and Col1), and the WH nail produced significantly higher bone mineral deposition and structural reconstruction in regenerated bone and callus region (Fig. 7, B and C). These findings underline the importance of finely tuned ion release and immune modulation for regeneration.

Within the callus, IL-4 and IFN-γ staining patterns were evaluated through the IL-4/IFN-γ ratio, which showed a distinct increase in the WH group compared with WHM and HAP. (Fig. 7D). CD11b/iNOS and CD11b/Arg1 double stains identified M1 and M2 macrophages (fig. S11, A and B). Notably, the WH group showed the lowest iNOS-positive area and the iNOS/Arg1 ratio (Fig. 7E), consistent with a reduced inflammatory burden and faster resolution of early recruited macrophages. TRAP staining demonstrated how ion release affected peri-implant osteoclastogenesis in osteoporotic bone. Although WHM released more Mg^2+^, it showed higher TRAP^+^ cell numbers than WH. This may be due to excessive Mg^2+^ stimulating IFN-γ–associated osteoclastogenic pathways (Fig. 7F). Hematoxylin and eosin (H&E) sections illustrated peri-implant marrow quality. The stainless nail formed a thin fibrotic capsule, and the PCL nail accumulated adipocyte-rich tissue. The calcium phosphate nails formed no fibrotic capsule, HAP retained immature blast-like cells, WH most resembled normal marrow, whereas WHM contained bony trabeculae with few marrow cells (Fig. 7G). After removal of the IMN, the refracture strength was measured, and the WH group exhibited a slightly higher mean value (fig. S11C).

There are concerns that Mg^2+^ may harm bone mineral integrity and quality (*28*, *29*). With the WH nail, Raman spectroscopy detected transient Mg-HAP (962 to 967 cm^−1^) and TCP (957 cm^−1^) peaks at day 7 around the implant site. By day 14, only the 962-cm^−1^ HAP remained, confirming that the WH nail preserves mature mineral integrity (fig. S12). These data indicate that a finely tuned, time-phased Mg^2+^ release from the WH-IMN aligns with the inflammatory phase–specific therapeutic window, curbs early T_H_1/M1-driven inflammation, supports T_H_2/M2-mediated repair, and preserves mineral integrity in osteoporotic fracture.

## DISCUSSION

Magnesium-rich biomaterials are widely reported to anti-inflammation and enhance osteogenesis. However, several studies also raise concerns that excessive Mg^2+^ can compromise bone mineral quality and exacerbate immune activation (*19*, *20*, *28*, *30*–*33*). This dual behavior could be particularly critical in osteoporotic bone, where the bone marrow microenvironment is characterized by chronic inflammation, elevated TNF-α and IL-1β, and a T_H_1/M1-biased immune profile (*1*, *2*, *4*, *5*, *34*). Therefore, because the effects of excessive Mg^2+^ can be further exaggerated in such a preinflamed environment, it is crucial to define an optimal Mg^2+^ concentration. Our study addresses this critical gap by delineating the Ca^2+^ signaling mechanisms affected by Mg^2+^ in CD4^+^ T cells, identifying stimulus-specific Ca^2+^ entry pathways (TRPM7, SOCE, and CaV) and engineering time-phased Mg^2+^-releasing IMNs that modulate the T_H_1/T_H_2 balance. Optimized WH IMN releases Mg^2+^ rapidly in early proinflammation to suppress T_H_1/M1 polarization, followed by a tapering phase that prevents late-phase proinflammation and supports T_H_2/M2-mediated tissue repair.

We have investigated the role of Mg^2+^ on CD4^+^ cells in response to TCR stimulation. Strong TCR/CD28 (3:1) stimulation triggers PLCγ1 to burst (*35*), and activating TRPM7 opens the channel completely (*36*), generating a rapid Ca^2+^ spike. TRPM7 is also a major gate for divalent cations and is inhibited by Mg^2+^ concentrations (*37*), and as extracellular Mg^2+^ rises into the millimolar range, it enters the membrane, competing with Ca^2+^ for the selectivity filter and suppressing the spike (*38*). Without this spike, calcineurin cannot efficiently dephosphorylate NFATc1. NFATc1 remains cytosolic, and T_H_1 program activation is reduced (*39*). Mg^2+^ (5 mM) with siTRPM7 did not delete the initial Ca^2+^ spike, confirming that the Mg^2+^ effect under strong stimulation is primarily TRPM7.

TCR stimulation evokes an initial ER Ca^2+^ release and a relatively slow second-phase increase in Ca^2+^ entry attributed to SOCE. Under moderate (1:1) stimulation, the Ca^2+^ fluorescence profile parallels that elicited by Tg-evoked tendency, siTRPM7 data indicate that TRPM7 contributes measurably only when the Ca^2+^ drive is strong enough to generate high-amplitude transients. It has been reported that when external Mg^2+^ is increased in millimolar, the Ca^2+^ influx rate is reduced by 20 to 40% (*40*) and alleviating excessive SOCE-induced ER stress with Mg^2+^ supplement (*35*). Our Ca^2+^ and Mg^2+^ imaging reveals that the elevated extracellular Mg^2+^ concentration drives Mg^2+^ influx through TRPM7 and Magt1 channels. After fast influx, high cytosolic Mg^2+^ level also triggers Mg^2+^ efflux for equilibrium, and a delayed Ca^2+^ peak emerges, which can be explained by the following mechanisms: Mg^2+^ transiently occupying divalent selectivity sites of ER pore and then vacating them with the ER stress gradient reverses as Mg^2+^ efflux. Also, Mg^2+^ does not directly occlude the CaV pore, and high extracellular Mg^2+^ can hyperpolarize the membrane and shift CaV channels below their activation threshold, consistent with our observations indicating Mg^2+^-driven ionic hyperpolarization (*41*, *42*).

When the TCR signal is weak, ER depletion is minimal and the membrane potential is maintained at about slightly above −50 mV (*43*). In low-stimulated situations where SOCE is insufficient, L-type CaV1.4 channels that have a low activation threshold that does not require strong depolarization can be opened (*44*). Under these conditions, the membrane potential regulating the ability of Mg^2+^ appears to be dominant. In summary, each stimulus layer passes the baton to a different Ca^2+^ gate (TRPM7-Orai1-CaV1.4), and Mg^2+^ regulates in a gate-specific manner, direct competition for TRPM7, Orai1 via SOCE stress attenuation, and voltage-dependent silencing of CaV. Without beads, Mg^2+^ markedly up-regulated STAT1 compared to STAT6. When we introduced an ATP-competitive JAK inhibitor, the Mg^2+^-driven increase in IFN-γ expression was abolished. These results imply that the extracellular rise in Mg^2+^ elevates the Mg·ATP, thereby enhancing JAK catalytic efficiency and tipping signaling dominance toward STAT1. These findings suggest that the rise in Mg·ATP preferentially boosts JAK-STAT1 signaling, giving STAT1 a functional advantage over STAT6.

NFAT isoforms are scattered throughout the cytoplasm but respond to distinct Ca^2+^ signaling (*45*, *46*). By modulating TCR strength with an anti-CD3/CD28 bead, we identified a two-step scheme: An early, high-amplitude Ca^2+^ spike through TRPM7 drives rapid nuclear entry of NFATc1 and skews CD4^+^ T cells toward T_H_1, whereas the waning stimulus yields a low-amplitude plateau carried by SOCE (Orai1) and CaV. This slower signal accumulates NFATc3, triggering a T_H_2 switch. Our results that TRPM7 loss weakens the first Ca^2+^ surge and NFATc1 import and prolonged Ca^2+^ keeps NFATc3 in the nucleus align with prior observations that loss of the initial spike and of NFATc1 nuclear translocation after TRPM7 blockade (*39*) and low-amplitude SOCE plateaus of Ca^2+^ maintain NFATc3 nuclear residency (*47*). These findings depart from most of the NFAT knockout literature, which frequently links NFATc1 to positive T_H_2 regulation and NFATc2/3 to T_H_1 control (*48*). A precise comparison is difficult because functional redundancy exists among NFAT isoforms (*49*), and our study supposes expression of all NFAT isoforms not knockout. There are also reports that indicate that constitutively active NFATc1 (caNFATc1) elevates IFN-γ while suppressing IL-4 in primary CD4^+^ T cells, reinforcing our observation that NFATc1 can act as a T_H_1 amplifier under certain signaling conditions. The precise mechanism behind these conflicting outcomes remains unresolved, and it is not clear whether the NFATc1/NFATc3 bias we observe is a direct consequence of Mg^2+^-driven activity or simply a context-dependent correlation. Determining this unconventional signaling hierarchy will require further targeted investigation. Also, Ca^2+^ signaling profiling distinguishes T_H_1, T_H_2, and T_H_17 lineages (*50*), yet little is known about how extracellular Mg^2+^ reshapes these patterns. Our data highlight this gap in T_H_1/T_H_2 and calls for follow-up work, particularly on Mg-modulated T_H_17 biology.

Although numerous studies have already studied that Mg^2+^ suppresses osteoclast differentiation (*10*), clear mechanism evidence is lacking. Our data suggest a mechanism based on Ca^2+^ flux observations. RANKL stimulates preosteoclasts with intermittent Ca^2+^ spikes. Then, Ca^2+^ spike activates calcineurin, dephosphorylates NFATc1, and pushes the cells toward fusion (*51*). We found that 5 mM extracellular Mg^2+^ entered through the TRPM7 and Magt1 channel, blunting the Ca^2+^ spikes, and inhibits TRAP-positive multinucleated cells. These effects were abolished by TRPM7 blockade, confirming channel specificity in Mg^2+^-Ca^2+^ relation. In our results, Mg^2+^ always reduces osteoclast differentiation in a concentration-dependent manner. This is thought to be because osteoclast differentiation is dominated by only NFATc1 (*52*, *53*). Since NFATc1 itself requires the Ca^2+^ spike that is lost by Mg^2+^, an increase in Mg^2+^ always pushes differentiation in the same downward direction. Therefore, an NFATc1 versus NFATc3 balancing act similar to the T_H_1/T_H_2 does not seem to exist. Furthermore, osteoclasts have a backdoor to Ca^2+^ signaling by TNF-α, IL-1, or other NF-κB–mediated cytokines (*54*), which Mg^2+^ cannot completely block likewise the WHM group in vivo. Also, osteoclasts can be increased due to secretion of RANKL by osteoblasts activated by Mg^2+^ (*55*). Further studies on the noncanonical or indirect pathways of Mg^2+^ in osteoclast differentiation are needed.

Macrophages have multiple Ca^2+^ pathways, including TRPM7, Orai1 (SOCE), transient receptor potential canonical 1 (TRPC1), and CaV (*56*). LPS activates TRPM7 and TRPC1 to induce M1 polarization, while Tg depletes SOCE and activates Orai1 to induce M2-specific genes (*57*–*59*). In both cases (LPS or Tg) of our experiments, Mg^2+^ enters through activated channels, inhibiting Ca^2+^ elevations and inhibiting both *iNOS* (M1) and *Arg-1* (M2) transcription. When LPS is applied, the Ca^2+^influx suppression effect of Mg^2+^ is confirmed to occur through TRPM7, similarly to other cells. Also, Tg that triggers Ca^2+^ flux by SOCE decreased in a Mg^2+^ concentration–dependent manner, and TRPM7 does not seem to be significantly involved in this process. Cytokine-only polarization (IFN-γ or IL-4) bypasses Ca^2+^ signal through, and Mg^2+^ no longer acts as a Ca^2+^ competitor, strengthens M1, and negatively affects M2. Our results are consistent with a recent study showing that (i) M1 macrophage polarization in calcium-stimulated culture environments, such as LPS-rich condition, is inhibited by Mg^2+^; (ii) higher Mg^2+^ entry up-regulates M1 in an IFN-γ environment, and low-dose Mg^2+^ (1 mM) flux via TRPM7 in an IL-4 environment supports phospho-signal transducer and activator of transcription 6 (pSTAT6) and increases M2 pool (*60*). We insist that both observations are correct and simply arise from different macrophage settings. Thus, the seemingly opposing results reflect cell condition–specific mechanisms: Mg^2+^ blocks the Ca^2+^-induced M1 pathway in LPS environments but shifts STAT signaling toward M1 in low-Ca^2+^ signal, cytokine-dominated environments. Moreover, prolonged exposure to high-Mg^2+^ levels feeds back to down-regulate TRPM7, reduces STAT6 phosphorylation, and ultimately collapses the M2 program. TRPM7 is expressed in T_H_1 > T_H_2, and high dose Mg^2+^ down-regulated Magt1 without significant changes in TRPM7 in CD4^+^ cells, but TRPM7-dependent kinase or homeostasis is required for macrophage M2 polarization, making sustained high Mg^2+^ unnecessary and detrimental for creating an M2 milieu. Therefore, harnessing Mg^2+^ for regeneration requires two complementary tactics: (i) establishing an IL-4–rich, IFN-γ–low environment that favors M2 activation from T_H_1/T_H_2 balance control and (ii) preventing the prolonged accumulation of excess Mg^2+^ during the late inflammatory phase.

Despite the significance of these findings, several limitations of the present study should be considered when interpreting the results. In this study, the ion-releasing ceramic materials were applied as coatings on stainless steel IMNs. Therefore, differences between the uncoated stainless steel control and the coated groups may partially reflect the presence of the coating itself, including potential differences in surface characteristics and physicochemical properties. In addition, although the coated groups shared the same substrate and overall implant design, the current experimental setting could not completely separate the effects of immune modulation from those of material- and mechanics-related factors during osteoporotic bone healing. Our findings suggest that controlled Mg^2+^ release shapes the osteoimmune microenvironment during healing, while the relative contribution of this immune regulation to the quality of repaired bone remains to be further clarified. Future studies incorporating more controlled mechanical conditions and direct correlation analyses between immune dynamics and bone repair outcomes will help further define these relationships.

Our findings find that Mg^2+^ blocks the Ca^2+^ flux at calcium stimulus conditions or activates the JAK-STAT1 pathway and shifts the T_H_1/T_H_2 and M1/M2 balance. This suggests that optimal amounts and a gradually decreasing release of Mg^2+^ are needed to a local bone immune environment in osteoporotic condition. WH IMN with a therapeutic Mg^2+^ window to maintain a T_H_2/M2-based environment through overall inflammatory phase enables precise regeneration of osteoporotic fractures. The finely tuned release of Mg^2+^ over the inflammatory period shows potential for a variety of orthopedic osteo-immunological applications.

## MATERIALS AND METHODS

### Material preparation

All reagents were analytical grade and purchased from Sigma-Aldrich or local distributors unless otherwise specified. WH [Ca_18_Mg_2_(HPO_4_)_2_(PO_4_)_12_] was synthesized by a wet precipitation protocol adapted from our previous work (*61*, *62*) or purchased product manufactured in the same way (OssDentix®, EGC Therapeutics, Republic of Korea). Briefly, 0.37 M Ca(OH)_2_ and 0.13 M Mg(OH)_2_ were mixed in deionized (DI) water under stirring and heated to 80°C. While maintaining this temperature, an equal volume of 0.5 M H_3_PO_4_ (in DI water) was added drop-wise (12.5 ml min^−1^). After 24 hours, the precipitate was collected, washed repeatedly with DI water, freeze-dried, and pulverized. Phase purity was confirmed by x-ray diffraction against the Joint Committee on Powder Diffraction Standards (JCPDS) reference pattern for WH (70-2064). HAP was prepared by the same route using 0.5 M Ca(OH)_2_ and 0.3 M H_3_PO_4_.

### Animal fracture and regeneration model

Animal tests were conducted following the guidelines for the Care and Use of Laboratory Animals by the Seoul National University (SNU-220526-2-1 and SNU-240521-11). Female BALB/c mice (8 weeks old) were anesthetized with alfaxan (100 mg kg^−1^, ip) and rompun (10 mg kg^−1^, ip). A mid-dorsal skin incision and bilateral muscle incisions exposed the ovaries. Each oviduct was ligated, the ovary was excised, and hemostasis was achieved with bipolar cautery. Animals were monitored for 8 weeks to establish the osteoporotic phenotype. Ovariectomized mice were reanesthetized with the same alfaxan/rompun mixture. After a lateral parapatellar incision, the patella was gently dislocated to expose the distal femur. The mid-diaphysis was transected with microscissors to create fracture. A 0.8-mm drill created an entry hole through the intercondylar notch into the medullary canal. IMNs fabricated from stainless steel, PCL, HAP, WH, or WHM were inserted to the fracture. Muscle and skin were closed in layers. Animals were housed, monitored daily, and euthanized at the designated endpoints by CO_2_ asphyxiation. Femora and marrow cells were harvested for subsequent analyses. The refracture test broke the bone and measured the breaking strength using a three-point bending test.

### FACS and T cell isolation and culture

Femora were excised from mice with or without fracture/IMN fixation; marrow cavities were flushed with ice-cold phosphate-buffered saline (PBS) to harvest bone marrow cells. For intracellular cytokine staining, 1 × 10^6^ cells were stimulated for 4 hours at 37°C with phorbol 12-myristate 13-acetate (50 ng ml^−1^), ionomycin (500 ng ml^−1^), brefeldin A (5 μg ml^−1^), and monensin (2 μM; all Thermo Fisher Scientific). Cells were then stained for 20 min at room temperature with the following antibody cocktail: anti-CD3 (11-0032-82, Thermo Fisher Scientific), anti-CD4 (12-0041-82, Thermo Fisher Scientific), anti-IFN-γ (11-7311-82, eBioscience), and anti–IL-4 (17-7041-82, eBioscience). For macrophage lineage analysis, separate aliquots were incubated for 20 min with anti-F4/80 (11-4801-82, Thermo Fisher Scientific), anti-CD80 (305207, BioLegend), and anti-CD206 (141707, BioLegend) followed by a wash with bovine serum albumin (BSA)–containing FACS buffer. Additional markers used in multicolor panels were anti-CD45 (103132, BioLegend), anti-Ly6C (128025, BioLegend), anti-Ly6G (127607, BioLegend), anti-B220 (103212, BioLegend), and anti-CD8α (47-0081-82, eBioscience). Data were acquired on a BD FACSAria II (BD Biosciences) installed at the National Center for Inter-university Research Facilities at Seoul National University and analyzed with FlowJo v10. CD4^+^ T cells were sorted from the same preparations for downstream Ca^2+^ imaging and differentiation assays. For in vitro experiments, cells were cultured in standard RPMI (Thermo Fisher Scientific) media supplemented with 10% fetal bovine serum, magnesium supplementation was added, the 1 mM Mg group received an additional 0.2 mM MgCl_2_, and the 2, 3, 4, and 5 mM groups received 1.2, 2.2, 3.2, and 4.2 mM MgCl_2_, respectively.

### Macrophage, osteoclast differentiation, and staining

Whole bone marrow cells from BALB/c mice were plated in α–minimum essential medium containing recombinant M-CSF (30 ng ml^−1^; Cell Signaling) for 3 days. Adherent BMMs were then cultured for 6 days in M-CSF (20 ng ml^−1^) plus RANKL (50 ng ml^−1^; Cell Signaling) to generate osteoclasts or in M-CSF (30 ng ml^−1^) for 7 days to obtain M0 macrophages for further experiments. For TRAP staining, a TRACP and ALP double stain kit (Takara Korea) was used. Briefly, after cultured osteoclast fixed 15 min in paraformaldehyde (PFA) 4% PBS, mixed solution of substrate for ACP and sodium tartrate with a 9:1 ratio was applied to fixed osteoclast. Two hours later, 50 μl of the supernatant was used to measure absorbance at 540 nm, and washed osteoclast was imaged with a microscope (Nikon Eclipse Ti2-E). Actin was stained with rhodamine phalloidin (Invitrogen) according to the manufacturer’s manual and imaged with a microscope. Using the ImageJ program, images were processed and analyzed. For the cell viability test, cells were stained with calcein AM/ethidium homodimer-1 solution. Live green and dead red fluorescence cells were imaged.

### Quantitative reverse transcription PCR and gene silencing

Total RNA was extracted from cultured cells with TRIzol (Thermo Fisher Scientific) following the manufacturer’s instructions. First-strand cDNA was synthesized from 1 μg of RNA using an M-MLV cDNA synthesis kit (Enzynomics, Korea). Quantitative polymerase chain reaction (PCR) was performed with TOPreal SYBR Green premix (Enzynomics) on a StepOne Real-Time PCR System (Applied Biosystems); primer pairs were designed to span exon-exon junctions and normalized to glyceraldehyde-3-phosphate dehydrogenase, and primer sequences are provided in the Supplementary Materials. Gene knockdown was achieved with predesigned pooled small interfering RNAs (Bioneer, Korea); cells were transfected with Lipofectamine RNAiMAX (Thermo Fisher Scientific) at 20 nM, and silencing efficiency was confirmed by quantitative reverse transcription PCR 48 hours posttransfection before downstream assays. For cocultured macrophages, because macrophages are adherent and T cells remain in suspension, nonadherent T cells were removed by gentle washing after coculture. RNA was then extracted directly from the remaining adherent macrophages without additional separation procedures.

### Calcium imaging

For Fura-2-AM ratiometric measurement, T cells, BMMs, pre-osteoclasts were washed and incubated in Fura solution [1 μM Fura-2-AM (Thermo Fisher Scientific), 0.05% (wt %) F-127, 1 mM probenecid (Sigma-Aldrich), and 10 mM Hepes in phenol red–free Hanks’ balanced salt solution] for 20 min in a 37°C incubator. With Infinite 200 pro microreader (Tecan), emissions were collected at 510 nm and excitation at 340/380 nm. The 340/380 ratios were calculated by a Δ*F*/*F*0 method. Also, cells were incubated 37°C, 20 min in Fluo-4 or Mag-Fluo-4 (Thermo Fisher Scientific) solution (same composition of Fura-2), and intensities were collected with constant time interval.

### IMN fabrication

Stainless IMNs were fabricated from stainless steel pins to Ø 0.8 mm by 12 mm. Stainless steel pins for the other IMNs were coated using a uniform PCL layering method (fig. S13) (*63*). Specifically, the pins were repeatedly dipped into a 10 wt % poly-ε-caprolactone (Sigma-Aldrich, 900825) solution in dichloromethane, with the dipping and pulling process repeated six times to achieve consistent coating. To control ion release, HAP, WH, and WHM powders were pretreated by sintering at 400°C for 4 hours in a muffle furnace (Jeiotech, Korea). For WHM IMNs, the powders were further modified by adding 10 wt % anhydrous MgPO_4_ before sintering, to increase the Mg/Ca ratio. The ceramic powder was then mixed with 5, 10, or 20 wt % in PCL solution and sequentially coated twice for a total of three coating steps, resulting in six immersion-pulling cycles onto the stainless steel pins while stirring. After coating, the pin surfaces were etched with NaOH to facilitate an initial burst release, followed by rinsing with deionized water.

### Histological analysis and immunostaining

The specimens (femurs) were fixed with 4% PFA and decalcified with 20% EDTA at pH 7.4. Specimens were dehydrated, paraffin-embedded, sectioned at 10 μm, and stained with H&E. For immunohistochemistry, deparaffinized sections were subjected to antigen retrieval (proteinase K), blocked with BSA, and incubated overnight at 4°C with primary antibodies. The signal was visualized with commercially available secondary fluorescence antibodies.

### SEM imaging

Samples were fixed on metal stubs with carbon tape and coated with platinum sputtering. Field emission scanning electron microscopy (SEM) (JSM-7800F Prime) was used to capture the microstructure and surface of the IMNs at 5 kV. Then, SEM images were analyzed with ImageJ software (National Institutes of Health, USA).

### Ion release measurement

Each IMN was immersed in distilled water and aged on the shaker for days at room temperature. Solutions were first centrifuged at 4000 rpm for 10 min and filtered through a membrane with 0.2-μm sized pores. Amounts of Ca and Mg ions in filtrates were measured with an inductively coupled plasma atomic emission spectrometer (OPTIMA 8300, PerkinElmer, USA) with argon plasma.

### Micro–computed tomography

The femur was scanned with the micro-CT scanner (Quantum GX2, PerkinElmer). The scanner settings were high-resolution 4 min, 0.5 mm Al + 0.06 mm Cu filter, 36-mm field of view, 4.5-μm voxel size resolution with automatically adjusted source energy, source current, and rotation step. Micro-CT software (CT Analyzer, Bruker, Belgium) was used for all analyses.

### Raman spectroscopy

Bone samples were deproteinized in 5% sodium hypochlorite to expose the mineral phase. Spectra were acquired with a DXR2xi confocal Raman microscope (Thermo Fisher Scientific) equipped with a 532-nm laser, a ×20 objective, and a 25-μm pinhole. The laser was focused on the fracture site; full-range spectra (100 to 3400 cm^−1^) were collected and baseline-corrected in OMNIC software.

### Statistical

Data were expressed as mean ± SD. Statistical analysis of the parametric data was performed using a one-way analysis of variance (ANOVA) with Tukey’s multiple comparisons tests. All plotting of data and statistical analyses were performed using GraphPad Prism 8.1 (GraphPad Software, La Jolla, CA, USA). *P* < 0.05 was considered statistically significant. Data were indicated with *P* values as ns (not significant), *P < 0.05, **P < 0.01, and ***P < 0.001.
