# Supplementary material for: Temporal immunomodulation of CD4+ T cells by magnesium regulates osteoimmune responses in osteoporotic fracture healing
Source: Sci Adv. 2026 Jun 26;12(26):eaeb2091. doi: 10.1126/sciadv.aeb2091 (PMC13308599; doi:10.1126/sciadv.aeb2091)
Supplement: Supplementary file 1 — Figs. S1 to S13 Table S1 [file sciadv.aeb2091_sm.pdf]

Science Advances
AAAS

Supplementary Materials for

Temporal immunomodulation of $CD4^+$ T cells by magnesium regulates osteoimmune responses in osteoporotic fracture healing

Jung Hun Kim *et al.*

Corresponding author: Nathaniel S. Hwang, nshwang@snu.ac.kr

Sci. Adv. **12**, eaeb2091 (2026)
DOI: 10.1126/sciadv.aeb2091

This PDF file includes:

Figs. S1 to S13
Table S1

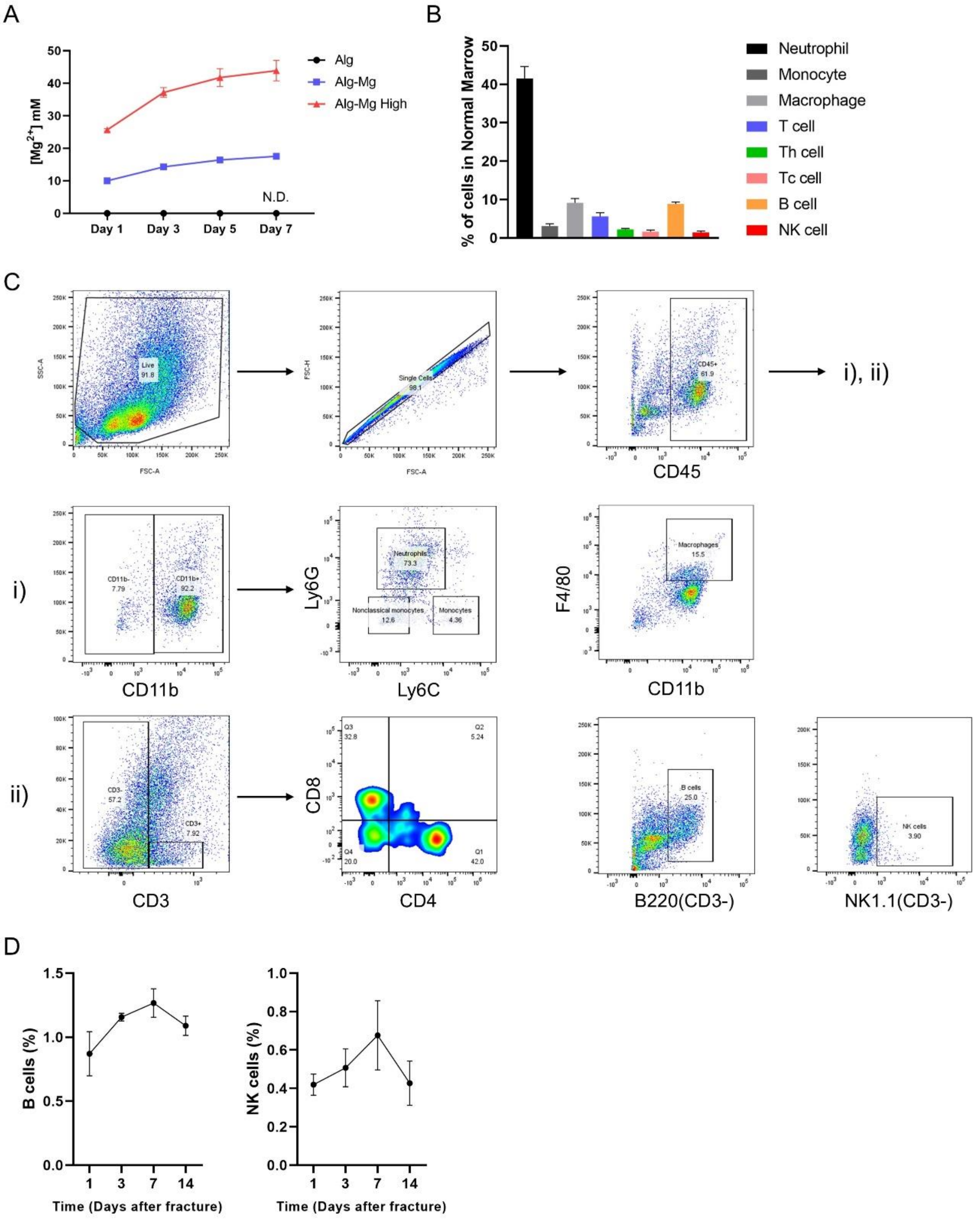

Fig. S1. Hydrogel Mg^{2+} release and Methodological overview of bone-marrow FACS profiling (A) Cumulative magnesium release curves for Alg-Mg and Alg-Mg High hydrogels. (B) Baseline distribution of major cell populations in balb/c mouse bone marrow determined by flow cytometry. (C) Flow-cytometry gating strategy used throughout the study, employing multiple antibody panels to resolve myeloid-lineage and lymphoid-lineage subsets. (D) Time-dependent changes in NK and B cell composition within the defect after hydrogel treatment (n=3).

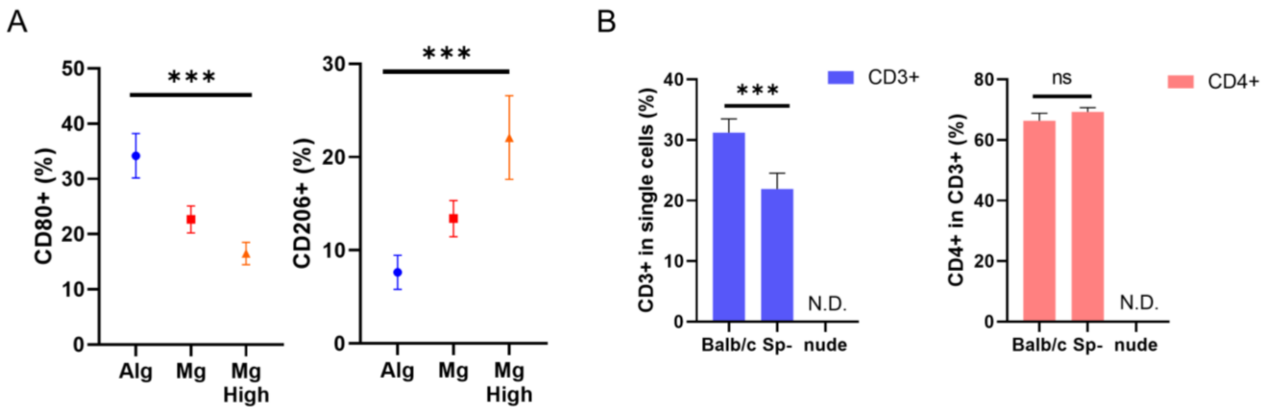

Fig. S2. Quantification of FACS analysis (A) Quantification of $CD80^+$ or $CD206^+$ cells as a percentage of $CD11b^+F4/80^+$ macrophages in balb/c at day 3 (n=3) (B) Quantification of $CD3^+$ and $CD4^+$ cells in blood at day 7 (n=6).

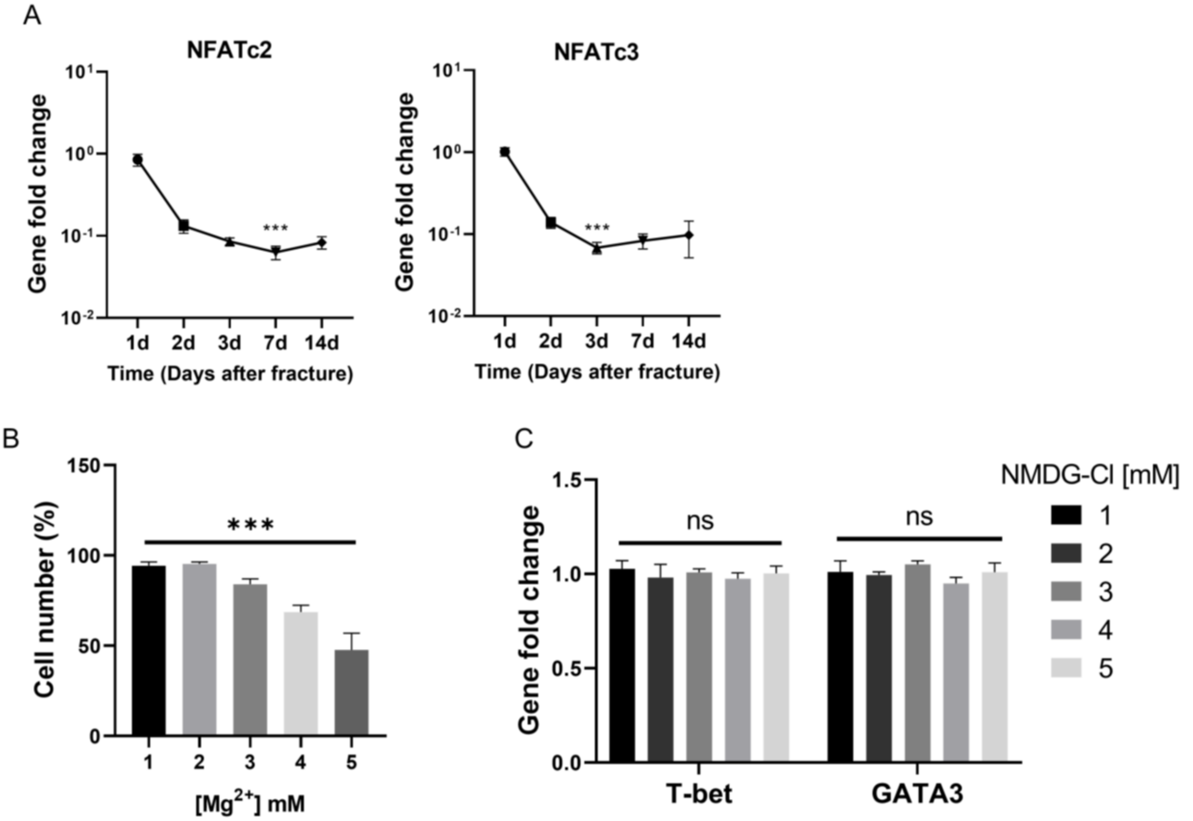

Fig. S3. NFATc expression in defect site and Mg^{2+}/NMDG-Cl effects on naïve $CD4^+$ T cells (A) Fold-changes of Nfatc2 and Nfatc3 transcripts in the bone-defect area (n = 3). (B) Relative cell numbers (%) of unstimulated $CD4^+$ T cells after 3-day culture in media containing the indicated extracellular Mg^{2+} concentrations (n = 3). (C) Relative mRNA levels of T-bet and Gata3 in $CD4^+$ T cells exposed to graded concentrations of N-methyl-D-glutamine chloride (NMDG-Cl) under 3:1 conditions (n = 3). Error bars denote mean ± SD; significance was assessed by Tukey's multiple-comparisons test (*p < 0.05; **p < 0.01; **p < 0.001).

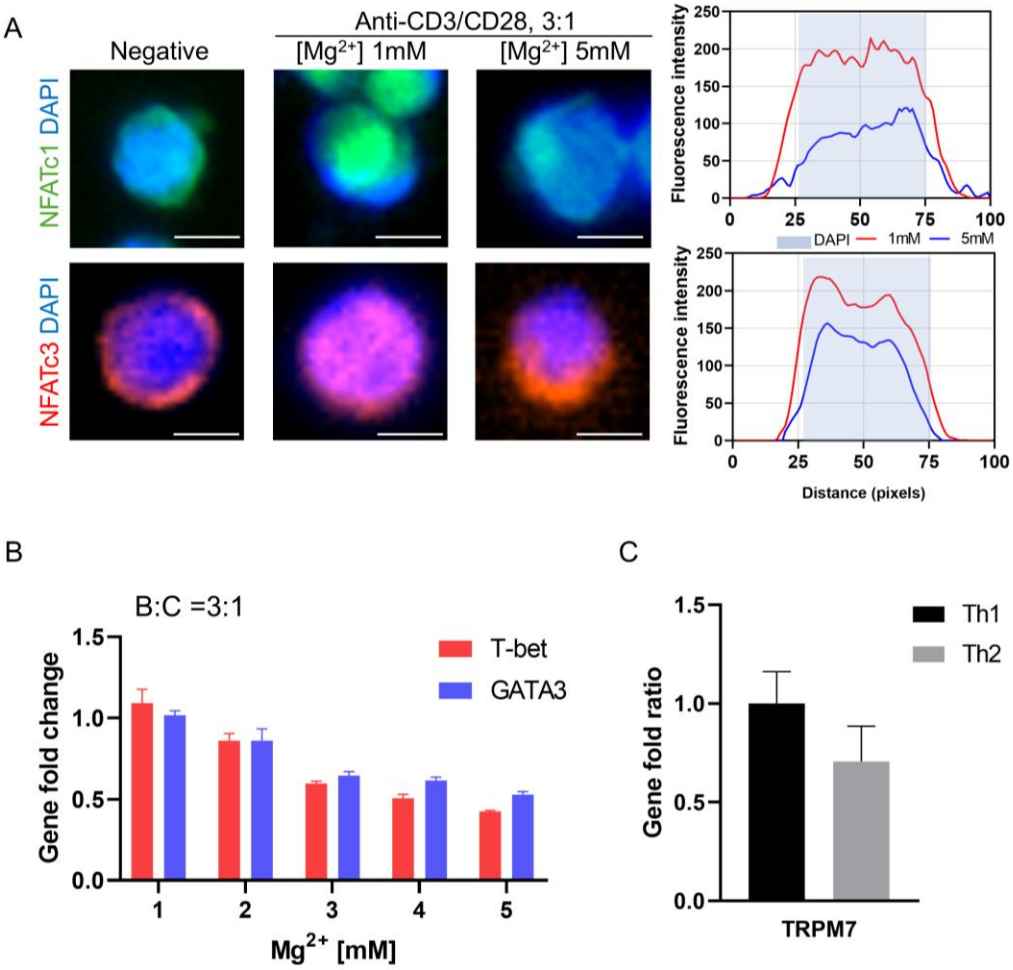

Fig. S4. Temporal NFAT transcript dynamics of CD4+ cell characteristics under strong stimulation (A) Peak Fura-2 Ca^{2+} fluorescence in CD4+ T cells exposed to the indicated extracellular Ca^{2+} concentrations (n = 3), (scale bar = 5 µm). (B) Immunofluorescence analysis of NFATc1 and NFATc3, representative images (left) and Linear intensity analysis NFAT nuclear localization. Fluorescent intensity was measured with a single x-y plane line trace of nuclear fluorescence intensity (right). (C) Expression of T-bet and GATA3 mRNA in CD4+ T cells stimulated at a B:C ratio of 3:1 across graded Mg^{2+} concentrations (n = 3). Error bars indicate mean ± SD; statistical significance was assessed by Tukey's multiple-comparisons test (*p < 0.05; **p < 0.01; **p < 0.001).

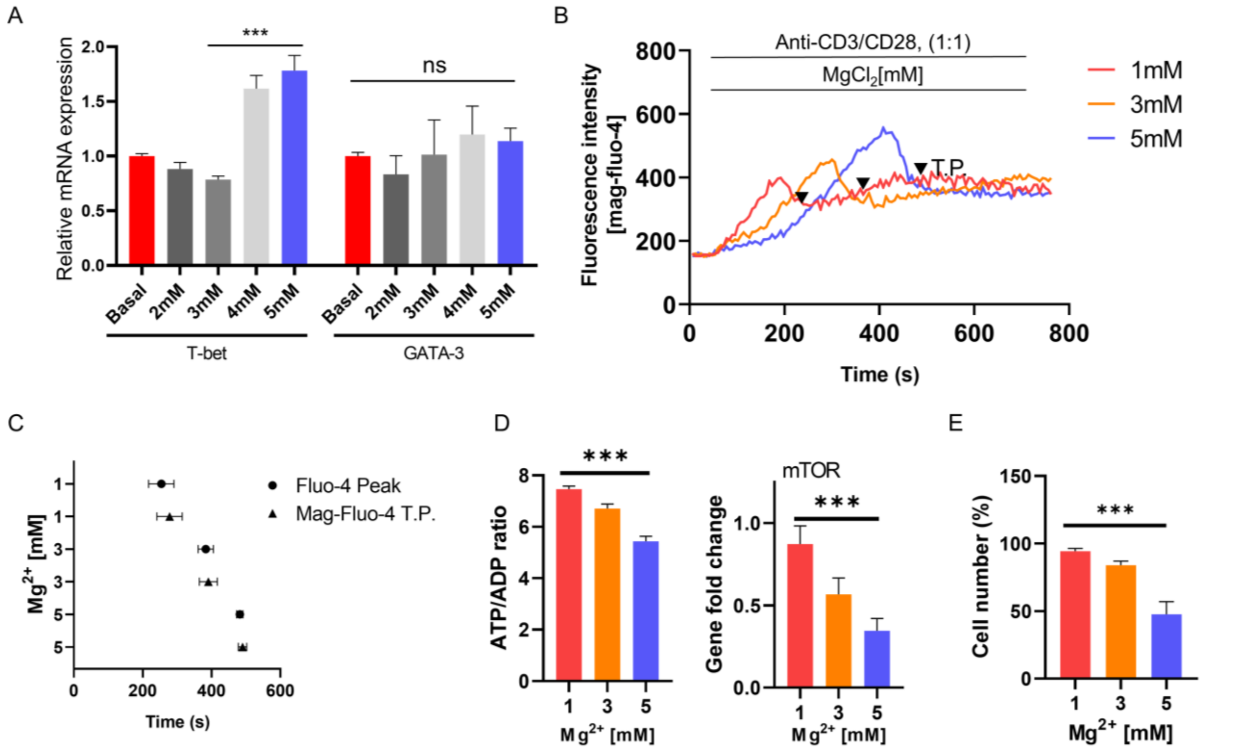

Fig. S5. Additional characteristics of CD4$^+$ T cells to extracellular Mg^{2+} with various stimulation intensity (A) Relative mRNA expression of T-bet and Gata3 after 3-day culture across graded Mg^{2+} with B:C=1:1. (B) Mag-Fluo-4 imaging of intracellular Mg^{2+} flux; T.P. = time point. (C) Time-aligned comparison of Fluo-4 Ca^{2+}-peak of Fig.3C amplitudes and Mag-fluo-4 T.P. of B (n = 3). (D) Cellular ATP/ADP ratios determined with a luminescence-based kit in no stimulation condition (n = 3). (E) Fold-change of mTOR mRNA under the no stimulation conditions (n = 3). (F) Percentage reduction in total cell number at increasing Mg^{2+} doses under no stimulation condition (n = 3). Error bars indicate mean ± SD. Statistical significance assessed by Tukey's multiple-comparisons test (*$p < 0.05$; **$p < 0.01$; **$p < 0.001$).

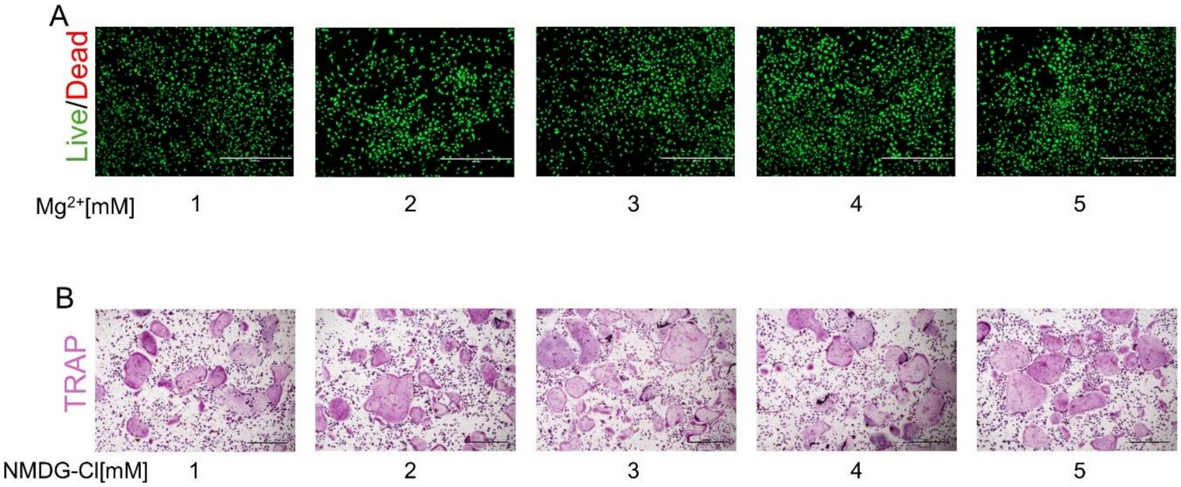

Fig. S6. **Viability of bone-marrow cells in Mg^{2+} and the impact of NMDG-Cl on osteoclastogenesis** (A) Representative Calcein-AM (live, green) and EthD-1 (dead, red) images of bone-marrow monocytes (BMMs) cultured 5 days in the indicated Mg^{2+} concentrations (scale bar = 400 µm). (B) TRAP staining of mature osteoclasts after 5 days with RANKL (100 ng ml^{-1}) plus escalating NMDG-Cl doses (scale bar = 500 µm).

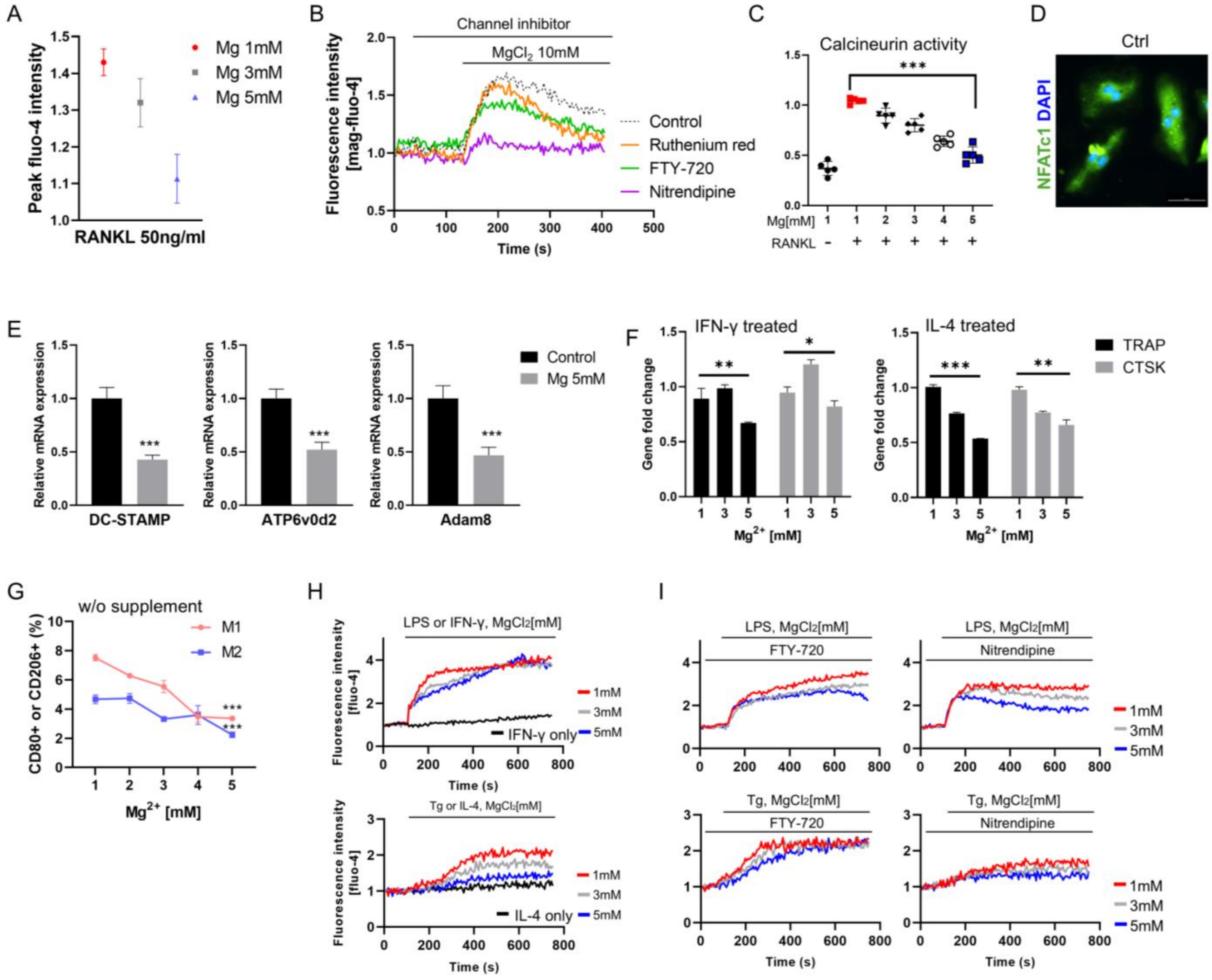

Fig. S7. Magnesium modulates RANKL-induced signaling in pre-osteoclasts and Ca^{2+} handling in macrophages (A) Distribution of Fluo-4 Ca^{2+}-peak amplitudes in pre-osteoclasts stimulated with RANKL (50 ng ml^{-1}) (n = 3). (B) Mag-Fluo-4 traces of intracellular Mg^{2+} in pre-osteoclasts, shown alone and with three individual channel inhibitors. (C) Calcineurin activity in pre-osteoclasts cultured with graded Mg^{2+}. (D) Immunofluorescence of NFATc1 in resting pre-osteoclasts (scale bar = 40 μm). (E) Expression of cell-fusion–related genes after Mg^{2+} treatment. (F) TRAP and CTSK mRNA levels when IFN-γ or IL-4 with Mg^{2+} during pre-osteoclast differentiation. (G) M1/M2 marker frequencies in unstimulated macrophages with varying Mg^{2+} (n = 3). (H) Representative Fluo-4 Ca^{2+} traces in M0 macrophages subjected to IFN-γ, LPS, thapsigargin (Tg), or IL-4. (I) Ca^{2+} imaging of LPS- or Tg-treated macrophages in the presence of a Mg^{2+}-transporter inhibitor. Error bars, where shown, indicate mean ± SD; significance was evaluated by Tukey's multiple-comparisons test (*p < 0.05; **p < 0.01; **p < 0.001).

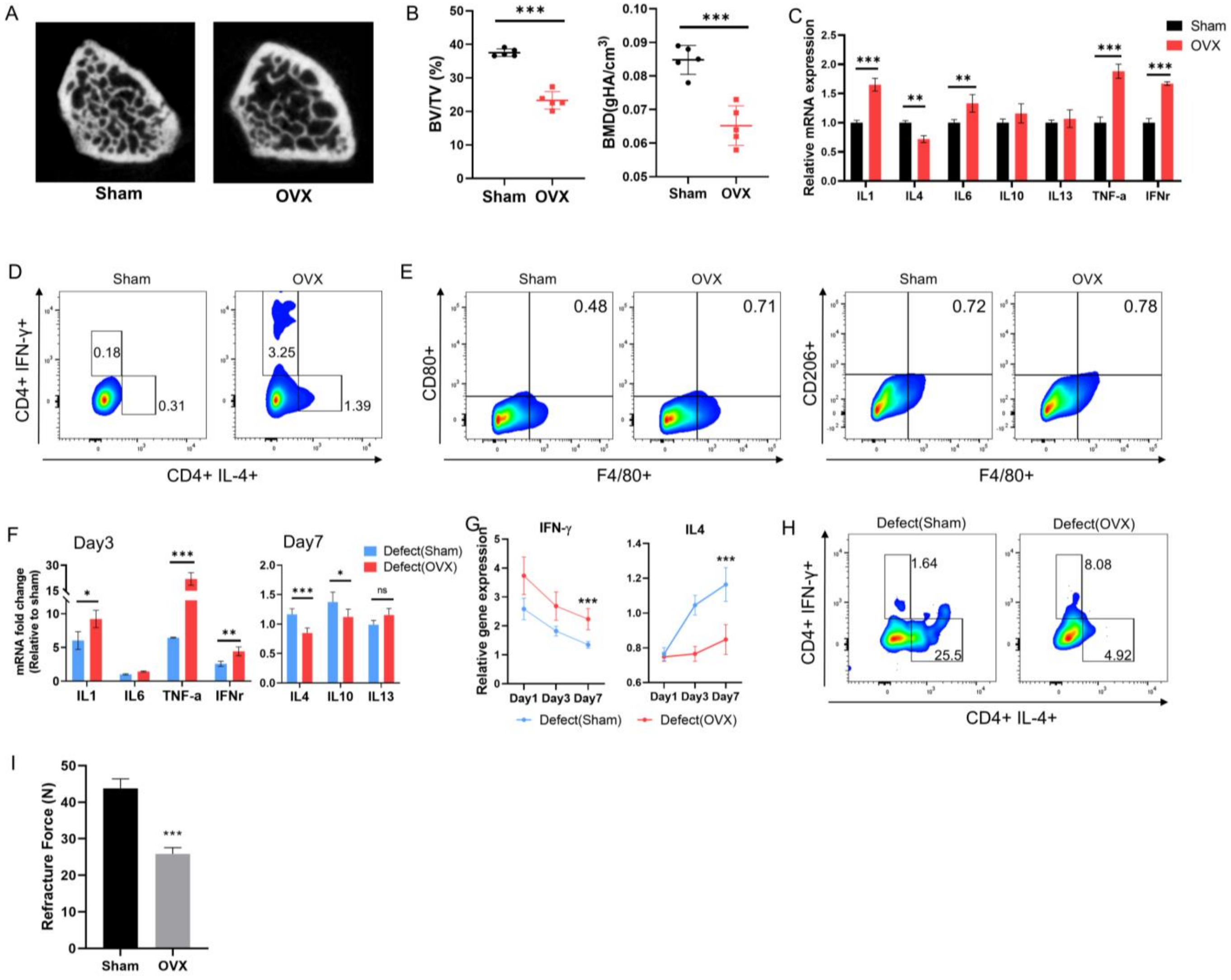

Fig. S8. Ovariectomy (OVX) exacerbates inflammatory bone loss and impairs regeneration (A) Micro-CT images of distal femora 8 weeks post-OVX in Balb/c mice. (B) Bone volume/total volume (BV/TV) and bone-mineral density (BMD) at week 8 (n = 5). (C) Expression of inflammatory genes in femoral bone-marrow cells from OVX mice (n = 3). (D) Flow-cytometry plots of $CD4^+IFN\text{-}\gamma^+$ and $CD4^+IL\text{-}4^+$ subsets in resting OVX bone-marrow cells. (E) Flow-cytometry plots of M1 and M2 macrophage in the resting OVX resting marrow. (F) Pro- and anti-inflammatory gene expression in marrow at day 1 and day 7 after femoral bone defect in sham versus OVX mice (n = 3). (G) Time-course of IFN-γ and IL-4 transcripts in defect tissue for sham and OVX group(n = 3). (H) Percentages of $IFN\text{-}\gamma^+$ and $IL\text{-}4^+$ cells in the hematoma at day 7 post-defect. (I) Three-point bending strength of regenerated femora at day 14 (n = 3). All quantitative data are presented as mean ± SD with Tukey's multiple-comparisons test for statistics (*p < 0.05; **p < 0.01; **p < 0.001).

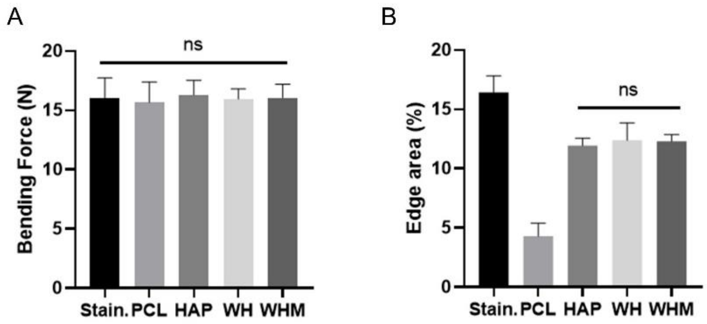

Fig. S9. Mechanical strength and topographical analysis of fabricated IMN (A) Average bending force of coated IMNs (B) Calculated edge area from IMN's SEM surface image with imageJ program

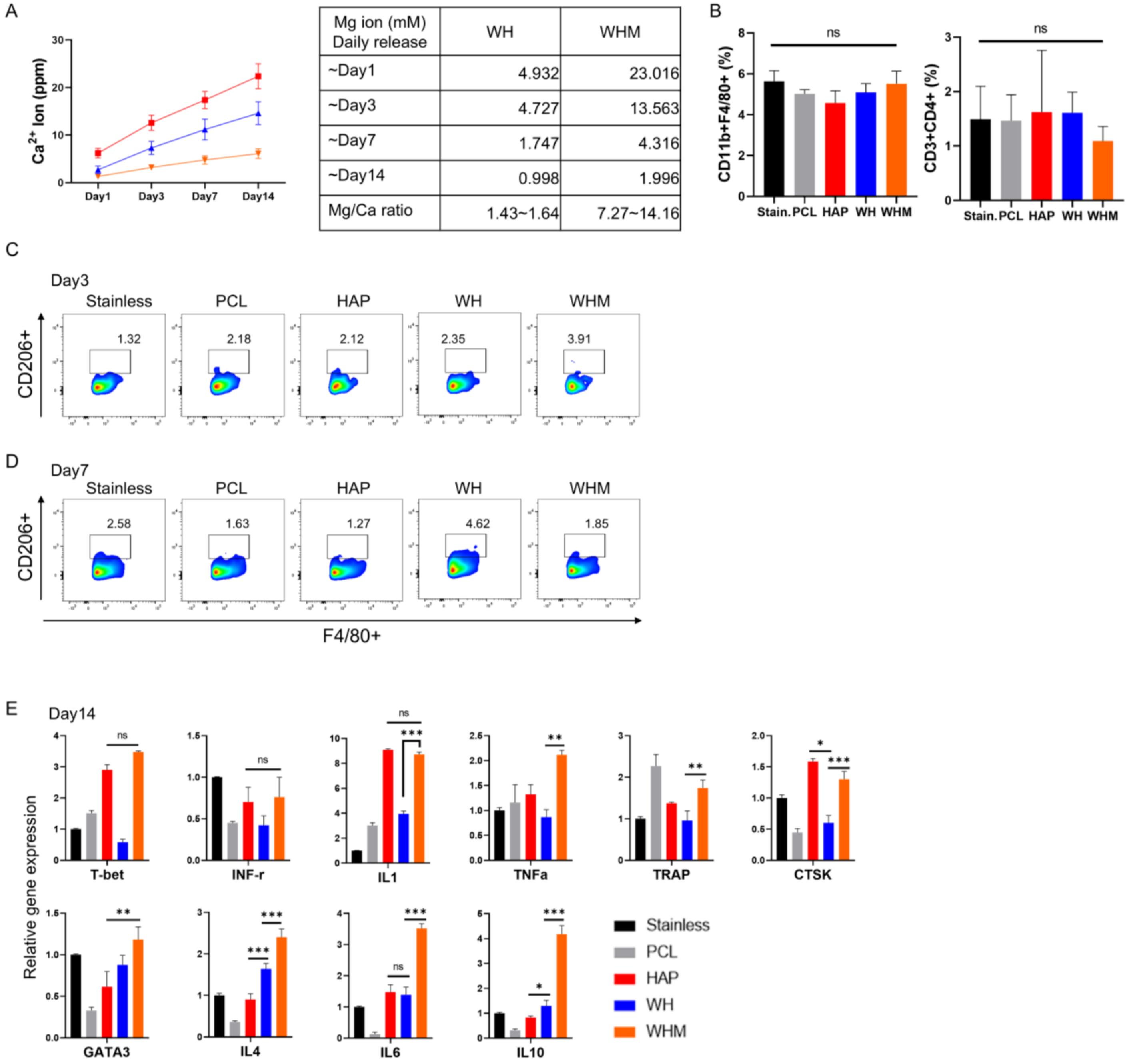

Mg ion (mM) Daily release	WH	WHM
~Day1	4.932	23.016
~Day3	4.727	13.563
~Day7	1.747	4.316
~Day14	0.998	1.996
Mg/Ca ratio	1.43~1.64	7.27~14.16

Fig. S10. Ion-release characteristics of IMNs and additional immunomodulatory data (A) Amount of Ca^{2+} released from each IMN (ppm), estimated daily Mg^{2+} release, and Mg/Ca release ratios (B) Proportion of CD11b+F4/80+ cells and CD3+CD4+ cells in early callus at day 3 (C, D) Representative FACS plots of M2 macrophages in bone marrow at day 3 (B) and day 7 (C). (E) Expression of inflammatory genes in defect tissue at day 14 (n = 3). Error bars indicate mean ± SD; Tukey′s multiple-comparisons test (*$p < 0.05$; **$p < 0.01$; **$p < 0.001$).

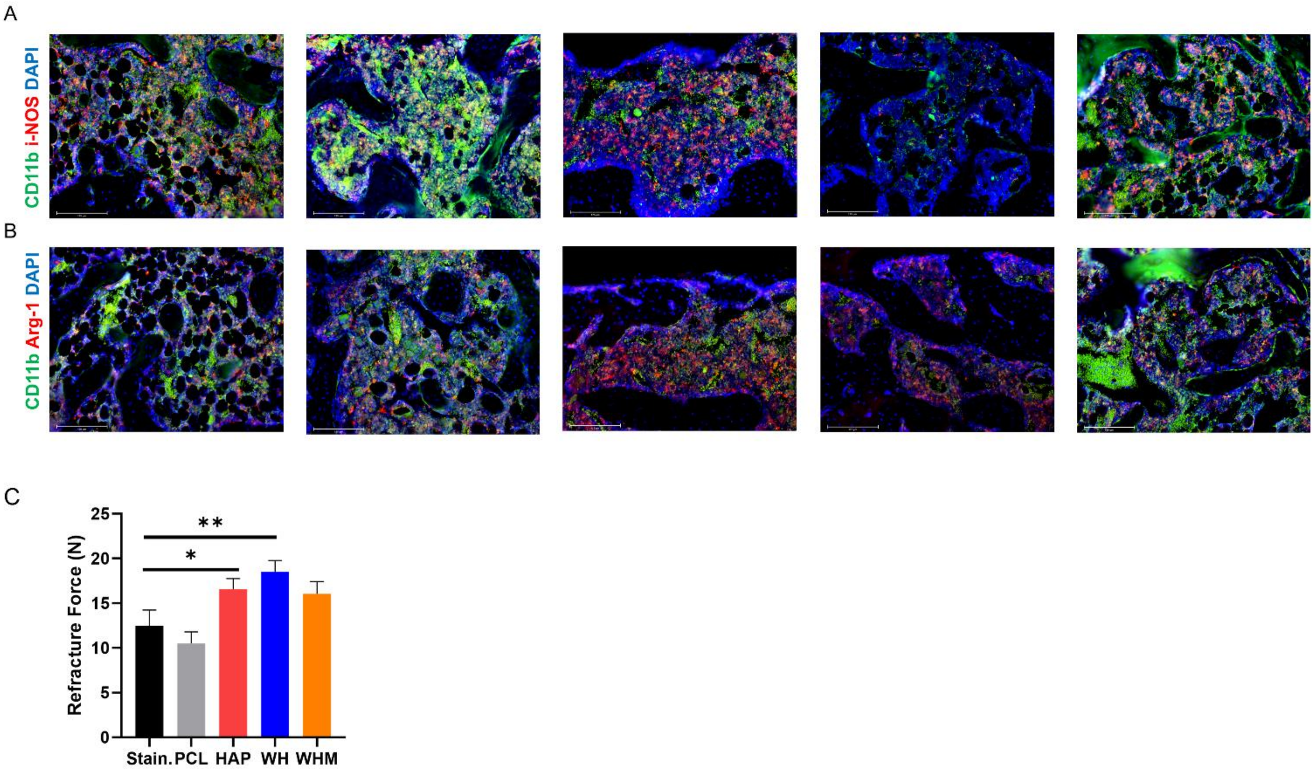

Fig. S11. Additional staining data and refracture force 4 weeks after fracture (A) CD11b/iNOS staining of the regenerated callus at 4 weeks; (B) CD11b/Arg-1 staining of the regenerated callus at 4 weeks. (C) Three-point bending strength of regenerated femora after IMN removal ($n = 3$). Error bars indicate mean ± SD; scale bar = 125 µm; Tukey′s multiple-comparisons test (*$p < 0.05$; **$p < 0.01$; **$p < 0.001$).

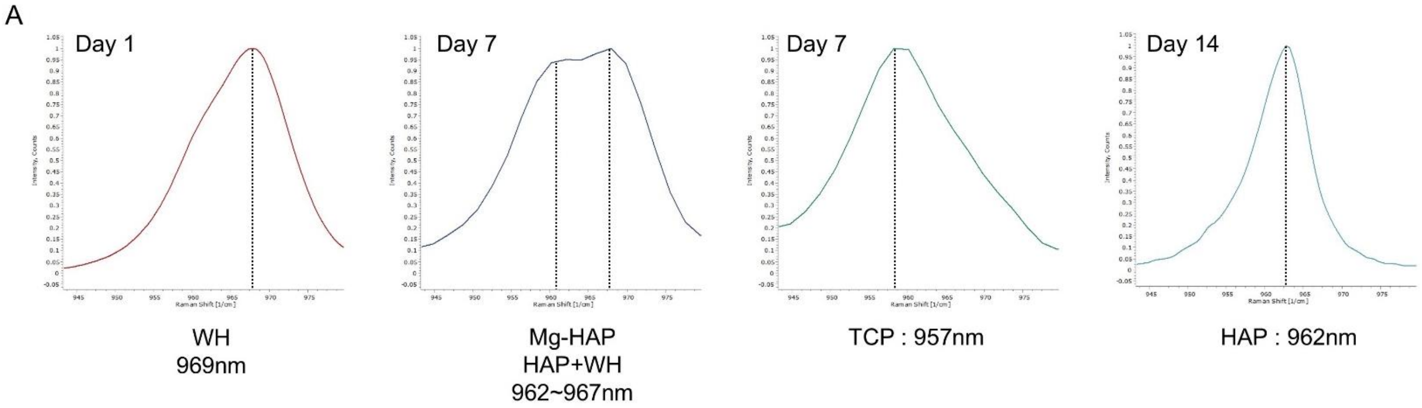

Fig. S12. Representative raman spectroscopy of nascent callus with characteristically observed peaks (A) Time-course Raman spectra of newly formed bone post-fracture; only hydroxyapatite peaks remain after day 14 (multiple sites in n=1 bone).

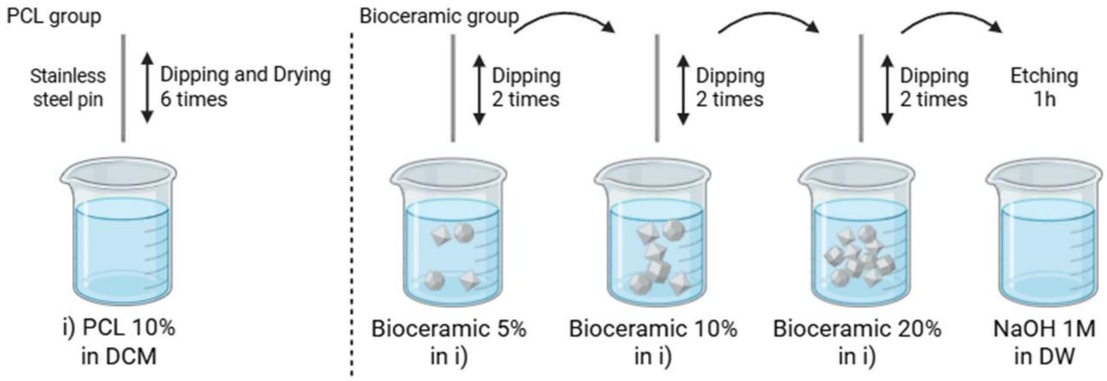

Fig. S13. IMN fabrication method scheme (Created in BioRender. Hwang, N. (2026) https://BioRender.com/v3uzxot)

Gene	Forward Primer	Reverse Primer
Adam8	TTGCCCCATGTGAAACAGTATG	AGGTGCAGGGTGAAAACGTG
Arg1	CTCCAAGCCAAAGTCCTTAGAG	AGGAGCTGTCATTAGGGACATC
ATP6v0d2	CAGAGCTGTACTTCAATGTGGAC	AGGTCTCACACTGCACTAGGT
CTSK	ACCCTTAGTCTTCCGCTCAC	CCCACATCCTGCTGTTGAGAA
DC-stamp	GGGGACTTATGTGTTTCCACG	ACAAAGCAACAGACTCCCAAAT
GATA3	CTCGGCCATTCGTACATGGAA	GGATACCTCTGCACCGTAGC
IFN-g	ATGAACGCTACACACTGCATC	CCATCCTTTTGCCAGTTCCTC
IL-1	GCAACTGTTCCTGAACTCAACT	ATCTTTTGGGGTCCGTCAACT
IL-10	GCT CTT ACT GAC TGG CAT GAG	CGC AGC TCT AGG AGC ATG TG
IL-13	CCTGGCTCTTGCTTGCCTT	GGTCTTGTGTGATGTTGCTCA
IL-4	GGTCTCAACCCCCAGCTAGT	GCCGATGATCTCTCTCAAGTGAT
IL-6	TAGTCCTTCCTACCCCAATTTCC	TTGGTCCTTAGCCACTCCTTC
iNOS	GTTCTCAGCCCAACAATACAAGA	GTGGACGGGTCGATGTCAC
IRF2	AATTCCAATACGATACCAGGGCT	GAGCGGAGCATCCTTTTCCA
IRF4	TCCGACAGTGGTTGATCGAC	CCTCACGATTGTAGTCCTGCTT
IRF5	GGTCAACGGGGAAAAGAAACT	CATCCACCCCTTCAGTGTACT
NFATc1	GGAGCGGAGAAACTTTGCG	GTGACACTAGGGGACACATAACT
NFATc2	TCATCCAACAACAGACTGCCC	GGGAGGGAGGTCCTGAAAACT
NFATc3	GCTCGACTTCAAACTCGTCTT	GATGTGGTAAGCCAAGGGATG
RELMa	CCAATCCAGCTAACTATCCCTCC	ACCCAGTAGCAGTCATCCCA
STAT1	TCACAGTGGTTCGAGCTTCAG	GCAAACGAGACATCATAGGCA
STAT6	CTCTGTGGGGCCTAATTTCCA	CATCTGAACCGACCAGGAACT
T-bet	AGCAAGGACGGCGAATGTT	GGGTGGACATATAAGCGGTTC
TNF-a	CCAGTGTGGGAAGCTGTCTT	AAGCAAAAGAGGAGGCAACA
TRAP	CACTCCCACCCTGAGATTTGT	CATCGTCTGCACGGTTCTG

Table 1. Primer sequence for RT-PCR
